# Endometrial scratching in women undergoing IVF/ICSI: an individual participant data meta-analysis

**DOI:** 10.1093/humupd/dmad014

**Published:** 2023-06-19

**Authors:** Nienke E van Hoogenhuijze, Gemma Lahoz Casarramona, Sarah Lensen, Cindy Farquhar, Mohan S Kamath, Aleyamma T Kunjummen, Nick Raine-Fenning, Sine Berntsen, Anja Pinborg, Shari Mackens, Zeynep Ozturk Inal, Ernest H Y Ng, Jennifer S M Mak, Sachin A Narvekar, Wellington P Martins, Mia Steengaard Olesen, Helen L Torrance, Ben W Mol, Marinus J C Eijkemans, Rui Wang, Frank J M Broekmans

**Affiliations:** Department of Gynaecology & Reproductive Medicine, University Medical Centre Utrecht, Utrecht University, Utrecht, the Netherlands; Department of Medicine, Utrecht University, Utrecht, the Netherlands; Department of Obstetrics and Gynaecology, University of Melbourne, Parkville, VIC, Australia; Department of Obstetrics and Gynaecology, University of Auckland, Auckland, New Zealand; Department of Reproductive Medicine, Christian Medical College, Vellore, Tamil Nadu, India; Department of Reproductive Medicine, Christian Medical College, Vellore, Tamil Nadu, India; Nurture Fertility, The Fertility Partnership, Nottingham, UK; School of Medicine, University of Nottingham, Nottingham, UK; Department of Obstetrics and Gynaecology, Fertility Clinic, Hvidovre, Copenhagen, Denmark; University Hospital Hvidovre, Hvidovre, Denmark; Fertility Clinic, Rigshospitalet, Copenhagen University Hospital, Copenhagen, Denmark; Centre for Reproductive Medicine, Universitair Ziekenhuis Brussel (UZ Brussel), Jette, Belgium; Department of Obstetrics, Konya Education and Research Hospital, Konya, Turkey; Department of Obstetrics and Gynecology, The University of Hong Kong, Queen Mary Hospital, Hong Kong SAR; Department of Obstetrics and Gynaecology, Assisted Reproduction Technology Unit, Prince of Wales Hospital, the Chinese University of Hong Kong 9F, Hong Kong SAR; Department of Reproductive Medicine, Bangalore Assisted Conception Center, Bangalore, Karnataka, India; Reproductive Medicine, SEMEAR Fertilidade, Ribeirão Preto, Brazil; Fertility Clinic, The Fertility Clinic Horsens Regional Hospital, Horsens, Denmark; Department of Gynaecology & Reproductive Medicine, University Medical Centre Utrecht, Utrecht University, Utrecht, the Netherlands; Department of Obstetrics and Gynaecology, Monash Medical Centre, Monash University, Clayton, VIC, Australia; School of Medicine, Medical Sciences and Nutrition, Aberdeen Centre for Women’s Health Research, University of Aberdeen, Aberdeen, UK; Department of Data Science and Biostatistics, Julius Center for Health Sciences and Primary Care, University Medical Centre Utrecht, Utrecht University, Utrecht, the Netherlands; Department of Obstetrics and Gynaecology, Monash Medical Centre, Monash University, Clayton, VIC, Australia; Department of Gynaecology & Reproductive Medicine, University Medical Centre Utrecht, Utrecht University, Utrecht, the Netherlands

**Keywords:** IPD, individual participant data meta-analysis, endometrial scratch, IVF, ICSI, frozen embryo transfer, ART, endometrial scratching, endometrial injury, endometrial sampling

## Abstract

**BACKGROUND:**

In IVF/ICSI treatment, the process of embryo implantation is the success rate-limiting step. Endometrial scratching has been suggested to improve this process, but it is unclear if this procedure increases the chance of implantation and live birth (LB) and, if so, for whom, and how the scratch should be performed.

**OBJECTIVE AND RATIONALE:**

This individual participant data meta-analysis (IPD-MA) aims to answer the question of whether endometrial scratching in women undergoing IVF/ICSI influences the chance of a LB, and whether this effect is different in specific subgroups of women. After its incidental discovery in 2000, endometrial scratching has been suggested to improve embryo implantation. Numerous randomized controlled trials (RCTs) have been conducted, showing contradicting results. Conventional meta-analyses were limited by high within- and between-study heterogeneity, small study samples, and a high risk of bias for many of the trials. Also, the data integrity of several trials have been questioned. Thus, despite numerous RCTs and a multitude of conventional meta-analyses, no conclusion on the clinical effectiveness of endometrial scratching could be drawn. An IPD-MA approach is able to overcome many of these problems because it allows for increased uniformity of outcome definitions, can filter out studies with data integrity concerns, enables a more precise estimation of the true treatment effect thanks to adjustment for participant characteristics and not having to make the assumptions necessary in conventional meta-analyses, and because it allows for subgroup analysis.

**SEARCH METHODS:**

A systematic literature search identified RCTs on endometrial scratching in women undergoing IVF/ICSI. Authors of eligible studies were invited to share original data for this IPD-MA. Studies were assessed for risk of bias (RoB) and integrity checks were performed. The primary outcome was LB, with a one-stage intention to treat (ITT) as the primary analysis. Secondary analyses included as treated (AT), and the subset of women that underwent an embryo transfer (AT+ET). Treatment-covariate interaction for specific participant characteristics was analyzed in AT+ET.

**OUTCOMES:**

Out of 37 published and 15 unpublished RCTs (7690 participants), 15 RCTs (14 published, one unpublished) shared data. After data integrity checks, we included 13 RCTs (12 published, one unpublished) representing 4112 participants. RoB was evaluated as ‘low’ for 10/13 RCTs. The one-stage ITT analysis for scratch versus no scratch/sham showed an improvement of LB rates (odds ratio (OR) 1.29 [95% CI 1.02–1.64]). AT, AT+ET, and low-RoB-sensitivity analyses yielded similar results (OR 1.22 [95% CI 0.96–1.54]; OR 1.25 [95% CI 0.99–1.57]; OR 1.26 [95% CI 1.03–1.55], respectively). Treatment-covariate interaction analysis showed no evidence of interaction with age, number of previous failed embryo transfers, treatment type, or infertility cause.

**WIDER IMPLICATIONS:**

This is the first meta-analysis based on IPD of more than 4000 participants, and it demonstrates that endometrial scratching may improve LB rates in women undergoing IVF/ICSI. Subgroup analysis for age, number of previous failed embryo transfers, treatment type, and infertility cause could not identify subgroups in which endometrial scratching performed better or worse. The timing of endometrial scratching may play a role in its effectiveness. The use of endometrial scratching in clinical practice should be considered with caution, meaning that patients should be properly counseled on the level of evidence and the uncertainties.

## Graphical abstract

**Figure:**
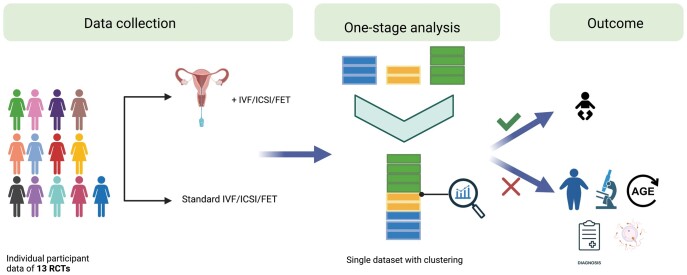
**Individual participant data-analysis shows that endometrial scratching in IVF/ICSI is likely to increase live birth rates; the effect did not differ between subgroups of women.** FET, frozen embryo transfer; RCT, randomized controlled trial. Created with BioRender.com.

## Introduction

Treatment with IVF/ICSI is the cornerstone of infertility treatment. It is estimated that each year approximately three million IVF/ICSI cycles are carried out globally (Chambers *et al.*, 2021). Despite advancements in many aspects of IVF/ICSI, the process of embryo implantation still seems to be the success rate-limiting step: pregnancy rates have remained stable at approximately 35% per embryo transfer in the past ∼5 years (De Geyter *et al.*, 2020).

Already early in the 20th century, it was discovered that in Guinea pigs endometrial decidualization could be induced by mechanical injury to the endometrium (Loeb, 1907). However, it took until the year 2000 for the incidental discovery that endometrial scratching could improve embryo implantation in women undergoing IVF/ICSI to take place, when Granot *et al.* (2000) published their findings that 11 out 12 women conceived after having undergone four sequential scratches prior to IVF/ICSI. Despite numerous randomized controlled trials (RCTs) on this topic, 20 years later it is still unclear if endometrial scratching affects live birth rates (LBR), and, if so, for which couples it could work, or how and when it should be applied. Hypotheses for the biological plausibility of scratching include that it would elicit an immune response or wound-healing response that is beneficial for embryo implantation, that it induces decidualization and thus aids in implantation, or that it would delay endometrial development of the hyperstimulated, advanced endometrium (Barash *et al.*, 2003; Li and Hao, 2009; Gnainsky *et al.*, 2015).

The lack of clarity might partly be caused by the fact that many RCTs were carried out on small samples and had a high risk of bias without data integrity checks, that there was a large heterogeneity in participant characteristics both within trials and between trials, and that the method and timing of scratching varied widely between studies (Li *et al.*, 2019; Lensen *et al.*, 2021). This has complicated conventional meta-analyses, leading to inconclusive results (Potdar *et al.*, 2012; Nastri *et al.*, 2015; Panagiotopoulou *et al.*, 2015). To overcome these problems, we performed an individual participant data meta-analysis (IPD-MA) on the topic. This IPD-MA aimed to answer the question of whether endometrial scratching in women undergoing IVF/ICSI alters the chance of a LB, and whether this effect is different in specific subgroups of women.

## Methods

This IPD-MA is an international collaboration of researchers that have been involved in RCTs that study endometrial scratching in women undergoing IVF/ICSI. The protocol for this IPD-MA was registered at PROSPERO (registration number: PROSPERO 2017 CRD42017079120) (van Hoogenhuijze *et al.*, 2017). The ethics committee of the University Medical Center Utrecht (MREC UMC Utrecht) judged that no ethics approval with regard to the Medical Research Involving Human Subjects Act (Dutch: WMO) was needed for the current study because the study participants were not subjected to any treatment or lifestyle or behavioral changes (MREC submission/response reference number WAG/mb/18/037685).

### Objective

The primary objective was to determine whether endometrial scratching alters the LBR in women undergoing IVF/ICSI, and whether specific participant characteristics modify the effect of scratching on the chance of LB (treatment-covariate interaction). The pre-specified participant characteristics of interest were age, treatment type [fresh embryo transfer versus frozen embryo transfer: (FET)], number of previously failed embryo transfers, cause of infertility, and timing of the scratch.

### Literature search

A systematic search and cross-referencing were performed in MEDLINE, EMBASE, and ClinicalTrials.gov to identify published and registered studies eligible for this IPD-MA. The search strategy is shown in Supplementary Table S1. The last systematic search was performed on 1 August 2018, after which automatic searches with alerts up until 1 September 2020 were used to identify new publications. In addition, all collaborators were asked if they knew of any other ongoing or published studies that were not included previously.

### Eligibility criteria

RCTs that compared endometrial scratching to a sham procedure or to no treatment in women undergoing IVF or ICSI with intended fresh or frozen embryo transfer with autologous oocytes were included in the IPD-MA. The scratch had to be performed with the intention to improve endometrial receptivity and could be performed by a biopsy catheter or curette. Non-intentional injury or endometrial disruption as a consequence of, for example, saline-infusion ultrasound were excluded. At least one of the outcomes of clinical pregnancy, ongoing pregnancy or LB had to be reported, and data had to be available before September 2020.

### Study selection and data collection

After the systematic search, all titles and abstracts were screened by two researchers (from N.v.H., S.L., W.M., E.H.Y.N., F.J.M.B.), after which two researchers (from S.L., E.H.Y.N., N.v.H., F.J.M.B.) assessed the remaining full-text papers/conference abstracts/registrations for eligibility. In case of doubts on eligibility criteria, the researchers were contacted through email. Remaining doubts and discrepancies were resolved with another reviewer (W.M. or H.T.).

Participation in this IPD-MA was requested by contacting the (corresponding) authors by email. In case of no response, the internet was searched for other email addresses of any of the authors, the institution was called by phone, authors were approached at congresses, and existing collaborators used their network to contact remaining authors.

After signing the Data Transfer Agreement, collaborators were asked to upload both their study protocol and anonymized data on a secured internet platform (SURFdrive). Data could be provided either as the original data file or transposed into the IPD-template, as an excel or SPSS document. Participating authors were offered co-authorship on this project pending sufficient contribution to the project according to the International Committee of Medical Journal Editors.

### Assessment of risk of bias and data integrity

All eligible studies of which a published or unpublished manuscript was available (collaborating and non-collaborating) were assessed for the risk of bias using the Cochrane Risk of Bias tool 2 (Sterne *et al.*, 2019) by two reviewers (N.v.H. and G.L.C.). Discrepancies were resolved by a third reviewer (R.W. or M.J.C.E.). The RCT in which N.v.H. was involved was assessed by two different reviewers (G.L.C. and R.W.).

Data integrity could only be assessed for collaborating studies and checks were carried out on several levels (Stewart *et al.*, 2015; Tierney *et al.*, 2015). First, all collaborators signed a Data Transfer Agreement in which they confirmed the authenticity of the data. Second, if an RCT was not registered prospectively and did not state having received ethics approval in the published manuscript, documentation of ethics approval was required. Third, during the data cleaning process all data were inspected for patterns and checked for missingness, distribution, outliers, protocol deviations, and ‘impossible combinations’ (e.g. not pregnant but did have a LB). Fourth, baseline and outcome tables from the publications were replicated for all studies. In case of discrepancies, the authors were contacted in order to explore differences.

### Data synthesis

All received data were transposed into a template using SPSS (IBM corp. Released 2017. IBM SPSS Statistics for Windows, version 25.0. Armonk, NY, USA: IBM Corp.) (SPSS, 2017), and individual participants were checked for inclusion and exclusion criteria (described above and in Supplementary Data File S1). In the process of transposing the data, uniformity of variable definitions was achieved through scrutinizing published manuscripts, trial registrations, provided study protocols, and by contacting the authors. Finally, all datasets were combined into one main dataset in SPSS, with an additional variable encoding the original trial to preserve clustering information.

### Statistical analysis

A statistical analysis plan (Supplementary Data File S1) with detailed information on the aims and statistical methods was drafted and agreed by the collaborator group prior to the start of data synthesis. Statistical analysis was performed using R Studio (version 1.2.5033) with R version 3.6.2 (R Core Team, 2019).

#### Primary, secondary, and *post hoc* analyses

The primary analysis included a one-stage, intention to treat (ITT) analysis for the outcome LB as well as a one-stage, as treated with embryo transfer (AT+ET) analysis for the interaction between endometrial scratching (yes/no) and the participant characteristics of interest (age; treatment type; number of previously failed embryo transfers; cause of infertility; timing of the scratch) and its effect on LB (Table 1). The interaction analysis was carried out in an AT+ET approach because the objective was to determine the true effect of participant characteristics on the effect size. This can best be done in a subset of the study sample that is evaluated according to the actual treatment received and with an actual chance of implantation (for which an embryo transfer is necessary).

**Table 1.** Overview of the types of outcomes, analysis, and statistical models used.

Type of outcome	Type of analysis	Model	Definition
**Primary outcome**			
Live birth	ITT	One-stage	Live birth from the first treatment after scratching
Participant characteristics *and* endometrial scratching *and* live birth	AT+ET; Interaction analysis	One-stage	Interaction between *endometrial scratching* and the following *participant characteristics*: age, treatment type, number of previously failed embryo transfers, and their effect on *Live birth*
**Secondary outcome**			
Live birth	ITT	Two-stage	Birth of a live fetus ≥24 weeks of gestational age after the cycle following randomization
Live birth	AT; AT+ET	One-stage; Two-stage	Birth of a live fetus ≥24 weeks of gestational age after the cycle following randomization
Ongoing pregnancy	ITT; AT; AT+ET	One-stage; Two-stage	Positive fetal heartbeat on ultrasound from a gestational age of ≥10 weeks after the cycle following randomization
Clinical pregnancy	ITT; AT; AT+ET	One-stage; Two-stage	Presence of a gestational sac on ultrasound at a gestational age of ≥6 weeks after the cycle following randomization
Multiple ongoing pregnancy	ITT; AT; AT+ET	One-stage; Two-stage	≥2 positive fetal heartbeats on ultrasound from a gestational age of ≥10 weeks after the cycle following randomization
Miscarriage	ITT; AT; AT+ET	One-stage; Two-stage	Loss of a clinical pregnancy. *Miscarriage rate:* number of women with a loss of a clinical pregnancy compared to the total number of women with a clinical pregnancy
Ectopic pregnancy	ITT; AT; AT+ET	One-stage; Two-stage	Participating center’s local definition of an ectopic pregnancy
Cumulative live birth*	ITT; AT; AT+ET	One-stage; Two-stage	Positive pregnancy test within 6 months after randomization, leading to the birth of a live fetus ≥24 weeks of gestational age
Pain	Descriptive		Patient-reported outcome on either a *Visual Analogue Scale (VAS)* or *Numeric Rating Scale (NRS)*
Adverse outcomes	Descriptive		Any adverse event reported by the study group

ITT, intention to treat; AT, as treated; AT+ET, as treated analysis on patients with an embryo transfer.

Overview of the planned primary and secondary outcomes with their definitions, the method of analysis (ITT, AT, or AT+ET) and the approach (one-stage or two-stage), as predefined in the statistical analysis plan (Supplementary Data File S1).

* This analysis was not performed due to too little follow-up data beyond the first transfer cycle.

Secondary analyses included (Table 1):

- one-stage ITT analyses for the other fertility outcomes (ongoing multiple pregnancy (OMP), ongoing pregnancy (OP), clinical pregnancy (CP), miscarriage, ectopic pregnancy, cumulative live birth of which the positive pregnancy test must be achieved within 6 months after randomization;
- one-stage AT analysis for all the pregnancy outcomes;
- one-stage AT+ET analysis for all the pregnancy outcomes;
- two-stage ITT, AT, and AT+ET analyses for all the pregnancy outcomes;
- descriptive analysis of pain and adverse outcomes after the scratch.

Additionally, publication and participating bias were evaluated by constructing a funnel plot. This analysis was *post hoc* extended by constructing forest plots of collaborating and non-collaborating RCTs. The analyses were further elaborated by two other *post hoc* analyses, namely a two-stage analysis of the interaction with age, and evaluation of the effect of the number of embryos that were transferred during the scratch-cycle.

#### Missing data

First, both sporadically and systematically missing data were imputed using multilevel multiple imputation of 45 imputations along 15 iterations according to a one-stage principle. Imputation was performed separately for the ITT and AT principle in order to take into account the differences in treatment variable (allocated versus received treatment) and consequently the treatment-covariate interactions (R packages used: mice version 3.12.0, micemd version 1.8.0, mitml version 0.4.3) (R Core Team, 2019) (Burgess *et al.*, 2013; Debray *et al.*, 2015; Jolani *et al.*, 2015).

#### Data synthesis

For all types of analysis, studies that did not report an outcome variable (i.e. outcome variable was missing for all participants) were excluded from the analysis of that variable, with the exception of OP, where many cases could be deduced from known data on CP and LB. Studies that did not report the actually received treatment on participant-level were excluded from all AT and AT+ET analyses but included in the ITT analysis, in which data on actually received treatment was imputed.

#### Two-stage meta-analysis

A two-stage analysis was performed in which the per-trial unadjusted odds ratios (OR) with 95% CI were obtained through a univariate logistic regression model for each pregnancy outcome. These per-trial estimates were then pooled using restricted maximum likelihood estimation and an inverse-variance weighted random-effects meta-analysis model, which allows for heterogeneity in the treatment effect. The 95% CI was calculated using a Student’s t-distribution and the Hartung–Knapp–Sidik–Jonkman correction (Inthout *et al.*, 2014).

#### One-stage meta-analysis

Univariate generalized linear mixed models were fitted for each outcome, according to the logistic model. To allow for between-study heterogeneity, random effects were used for both the intercept and the slope. As this analysis is hierarchical, the studies are weighted according to the sample size in the meta-analysis. In this first model, we obtained the unadjusted ORs and 95% CIs for scratching versus control. We subsequently extended the models to include fixed-effect adjustment terms for participant age, BMI, cause of infertility, duration of infertility, number of previously failed embryo transfers, and treatment type (no, fresh, or frozen embryo transfer), to obtain the adjusted ORs with 95% CIs.

The choice for adjustment factors was based on several factors. First, from a statistical point of view, the interaction variables need to be included. Second, we aimed to include variables that are known to impact the chance of pregnancy/LB. Third, the complexity of the imputation model was dependent on the availability of data (this limited, for example, adjustment for embryo quality). With too many adjustment variables, our model became overly complex, leading to overparametrization problems. Additionally, for clinical purposes it would be most useful to include factors that are known at the time when a physician decides to offer a patient scratching or not (e.g. embryo quality is not known at that time).

In our IPD population we observed that a relatively high number of participants had received three or more embryos in their study-transfer, while this is not commonly advised practice anymore. To evaluate whether the number of transferred embryos could be related to the scratch effect, we decided to conduct a *post-hoc* one-stage analysis with adjustment for the number of transferred embryos.

To account for possible bias in the study design or performance, we conducted a sensitivity analysis by repeating the one-stage unadjusted and adjusted analysis separately for studies with a low risk of bias, and for the studies with a low or medium risk of bias.

#### Subgroup analysis (treatment–covariate interaction)

The interactions between endometrial scratching and participant characteristics were determined for the outcome LB in a one-stage model, in the AT+ET subset only. We excluded the registered trial by Lee, because interaction analysis in a very small sample (n = 15) is unreliable and can thus distort the overall result (Lee, n.d.). We pre-specified two categorical interaction variables: cause of infertility (male factor versus unexplained infertility versus other causes) and treatment type (no embryo transfer versus fresh embryo transfer versus frozen embryo transfer), and two continuous interaction variables: age and number of previously failed embryo transfers. We were unable to calculate the fifth interaction of interest, the effect of timing of the scratch on its effect on live birth, owing to too few IPD on the within-cycle timing and too little within-trial variability in timing (i.e. almost all studies applied the same timing to their full sample).

Each interaction was evaluated separately by extending the one-stage adjusted model with the interaction term(s). In order to separate within-trial and between-trial information, we centered the covariates about the study means, and included the interaction terms with the study means of the covariate (Riley *et al.*, 2020). We thus analyzed both the within-trial and between-trial interaction in each interaction analysis. For the continuous interaction terms, this resulted directly in a *P*-value describing the interaction. For the categorical interaction terms, the models with and without the interaction variable were compared using the likelihood ratio test (R packages used: mice version 3.12.0, function D3), which then resulted in a *P*-value for the interaction (R Core Team, 2019). In addition, for the continuous variables, we plotted the logOR and 95% CIs as a function of age and number of previous failed transfers, respectively.

#### Descriptive analysis

Participant characteristics and the post-randomization trajectory were presented using descriptive analysis in which the median and interquartile range (IQR) or the number and percentage were calculated based on the imputed dataset. The outcomes of pain directly after the scratch (measured on a visual analogue scale or on a numeric rating scale) and adverse events in the week following the scratch were generally only recorded for the intervention group. We therefore reported these outcomes as a mean with SD (pain) and as the number of events (adverse events). Missing data for pain and adverse events were not imputed.

#### Evaluation of publication bias and participation bias

Publication bias was analyzed according to standard meta-analysis methods by constructing a funnel plot based on the two-stage ITT outcomes for LB (Sterne and Egger, 2001). Both participating and non-participating studies were included.

As not all authors of RCTs agreed to collaborate, we intended to evaluate whether a systematic difference in risk of bias and outcome was present between IPD and aggregate data (AD) studies. We therefore assessed all published studies for risk of bias using the Cochrane Risk of Bias Tool 2 (Sterne *et al.*, 2019), and performed a meta-analysis to visualize in a forest plot whether the pooled effects of IPD (based on the two-stage meta-analysis) and of AD studies (who did not share IPD, based on conventional meta-analysis) were different.

## Results

### Studies and participants

**Figure 1.**
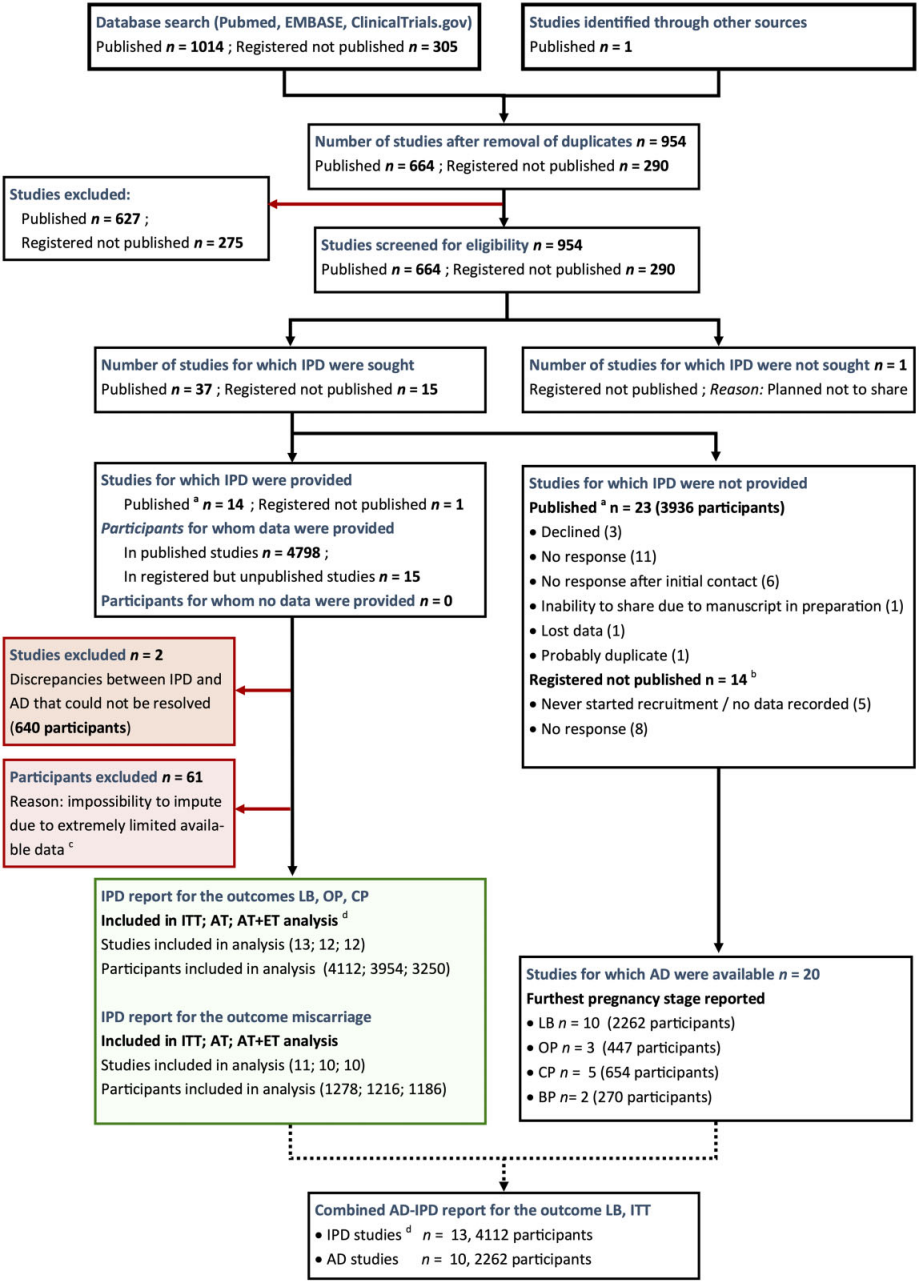
**Flowchart of the systematic literature search, screening, and study selection.** Flow chart describing the systematic search for both published (full articles and conference abstracts) and unpublished trials, and subsequent study selection. ^a^Published as full paper or as a conference abstract. ^b^Number of participants unknown because it is unclear if/how many participants have been included. ^c^Limited available data due to various reasons, a.o. closure of one of the centers or withdrawal directly after randomization. ^d^13 studies, of which 11 published as full text, 1 published as conference abstract, 1 registered but unpublished. n, number; IPD, individual participant data; AD, aggregate data; ITT, intention to treat; AT, as treated; AT+ET, as treated with embryo transfer; LB, live birth; OP, ongoing pregnancy; CP, clinical pregnancy; BP, biochemical pregnancy.

Our systematic search resulted in 52 eligible studies, of which 15 study groups comprising 4813 participants agreed to share IPD (refer to Fig. 1 for the full flow chart and Supplementary Table S2 for the list of non-participating studies). Compared to a total of 7690 reported participants, this equates to an inclusion rate of 63%. The authors of one additional trial agreed to share IPD in the future, as their main publication and secondary papers were still in preparation (Metwally *et al.*, 2020). Two RCTs from which IPDs were obtained were excluded after data integrity checks owing to discrepancies between the received IPD and the published outcomes that could not be resolved despite email contact with the authors (Mahran *et al.*, 2016; Hebeisha *et al.*, 2018). From the remaining 13 RCTs, a few individual cases were excluded from analysis as no data were available at all (making it impossible to impute data), which could be due, for example, to immediate withdrawal of consent or closure of a participating center (Yeung *et al.*, 2014; Polanski *et al.*, 2015; Berntsen *et al.*, 2020). This resulted in final inclusion of 13 RCTs comprising a total of 4112 included participants, equating to an inclusion rate of 58% (subtracting the two excluded trials from a total of 7690 participants leaves data on 4112/7050 reported participants’ data).

Of note, several study groups agreed to collaborate but subsequently failed to respond despite multiple attempts of contact (Supplementary Table S2). Study groups were approached via email (multiple times), LinkedIn, telephone contact with their institute and ‘live’ at congresses—all of the participating authors contributed to this so as to optimize participation. This process has been extended up to the last moment of compiling the dataset, which all in all took over 3 years, thereby providing sufficient time for eligible study groups to respond.

All studies provided data on CP and LB, 11 studies also provided data on OP and for the other two studies most OPs could be deduced from CP and LB data (Nastri *et al.*, 2013; Polanski *et al.*, 2015). As we uniformized the definitions for all pregnancy outcomes, data presented in this IPD may differ from data of the original publications (Yeung *et al.*, 2014; Polanski *et al.*, 2015; Lensen *et al.*, 2019; Berntsen *et al.*, 2020). For example, Lensen *et al.* (2019) report in their original paper all LBs resulting from the first embryo transfer that was carried out, given that this was within 3 months after randomization. In contrast, this IPD included LBs resulting from the first treatment after randomization. Differences may thus arise when after randomization a participant starts a fresh IVF/ICSI cycle followed by a ‘freeze all’, and a FET 2 months after treatment from which she conceives. In the original paper this would have been regarded as a pregnancy, while in the IPD this would have been no pregnancy due to no embryo transfer. Other causes of discrepant inclusion numbers are when participants withdrew consent immediately after randomization, resulting in no available data at all and making imputation impossible, or when patients were included incorrectly (i.e. did not meet inclusion criteria). Data on miscarriages were provided by 12 studies (all but Narvekar), and on ongoing multiple pregnancy by 11 RCTs (all but Narvekar and Mak) (Narvekar *et al.*, 2010; Mak *et al.*, 2017). One trial did not record which participants actually underwent an endometrial scratch and embryo transfer, and was thus excluded from the AT and AT+ET analyses (Nastri *et al.*, 2013). Only two trials systematically recorded outcome data beyond the study-cycle embryo transfer, and therefore the planned analysis of 6-month pregnancy leading to LB—as described in the study protocol—was dropped (van Hoogenhuijze *et al.*, 2017, 2020; Mackens *et al.*, 2020).

Nine RCTs studied scratching in participants undergoing fresh IVF/ICSI, two in fresh or frozen embryo transfer (Polanski *et al.*, 2015; Lensen *et al.*, 2019), and two in participants undergoing FET only (Lee, n.d.; Mak *et al.*, 2017). All but one performed the scratch using a biopsy catheter; Berntsen used hysteroscopic forceps (Berntsen *et al.*, 2020). Four trials applied a non-intracavitary sham procedure (Lee, n.d.; Nastri *et al.*, 2013; Polanski *et al.*, 2015; Mak *et al.*, 2017). Further study characteristics are listed in Table 2.

**Table 2.** Design features and demographic characteristics of included RCTs.

1st author, year	Published, conference abstract or registered only	Country	Recruitment period	Type of treatment	Study population	Device used for scratching	Comparator	Furthest stage of pregnancy reported as outcome	Number of participants	Mean female age in years (range)	Mean female BMI in kg/m^2^ (range)	Mean duration of infertility in months (range)	Mean number of failed embryo transfers (range)
**Narvekar** 2010	Published	India	May 2007–Jul 2008	IVF/ICSI	Undergoing IVF, at least one previous failed cycle	Biopsy catheter	No intervention	LB	99	32.2 (23–37)	25.5 (18–34)	NA	NA
**İnal** 2012	Published	Turkey	January 2008–March 2009	ICSI	Undergoing IVF, at least one previous failed cycle	Biopsy catheter	No intervention	LB	100	30.3 (24–38)	24.0 (19–32)	28.0 (14–44)	1.4 (1–3)
**Nastri** 2013	Published	Brazil	June 2010–March 2012	IVF/ICSI	Undergoing IVF	Biopsy catheter	Drying of the cervix	LB	158	32.3 (23–38)	NA	NA	1.6 (0–3)
Yeung 2014	Published	Hong Kong	March 2011–October 2013	IVF/ICSI	Undergoing IVF	Biopsy catheter	No intervention	LB	300	36.1 (27–45)	22.2 (15–32)	55.7 (6–204)	0.6 (0–6)
Raine-Fenning 2015*	Conference abstract	UK	January 2013–January 2015	IVF/ICSI/FET	Undergoing IVF or FET	Biopsy catheter	Transvaginal ultrasound	LB	160	32.9 (24–43)	24.8 (14–35)	39.2 (12–144)	0.5 (0–5)
**Mak** 2017	Published	Hong Kong		FET	Undergoing FET	Biopsy catheter	Endocervical manipulation	LB	186	36.3 (27–44)	21.6 (10–30)	55.8 (2–241)	1.7 (0–9)
**Aleyamma** 2017	Published	India	April 2008–April 2015	IVF/ICSI	Undergoing IVF, at least one previous failed cycle	Biopsy catheter	No intervention	LB	111	31.8 (23–38)	25.1 (14–37)	NA	1.5 (1–6)
**Lensen** 2019	Published	NZ, UK, Belgium, Sweden, Australia	June 2014–June 2017	IVF/ICSI/FET	Undergoing IVF or FET	Biopsy catheter	No intervention	LB	1364	34.9 (23–46)	24.7 (16–58)	49.8 (0–200)	1.2 (0–11)
**Olesen** 2019	Published	Denmark	February 2014–December 2017	IVF/ICSI	Undergoing IVF, at least one previous failed cycle	Biopsy catheter	No intervention	LB	304	31.9 (21–40)	24.0 (17–39)	NA	2.4 (1–8)
**Mackens** 2020	Published	Belgium	April 2014–October 2017	IVF/ICSI	Undergoing IVF	Biopsy catheter	No intervention	LB	200	33.2 (22–39)	23.3 (17–34)	NA	NA
**Berntsen** 2020	Published	Denmark	2013–2018	IVF/ICSI	Undergoing IVF, at least one previous failed cycle	hysteroscopic forceps	No intervention	LB	229	34.3 (22–40)	24.1 (17–37)	NA	1.2 (1–4)¹
**Van Hoogenhuijze** 2020	Published	Netherlands	January 2016–August 2019	IVF/ICSI	Undergoing IVF, one previous failed cycle	Biopsy catheter	No intervention	LB	933	35.1 (22–43)	24.6 (17–42)	34.6 (1–240)	2.2 (1–12)
**Lee**	Registered	Hong Kong	2012–2013	FET	Undergoing FET, no pregnancy in previous fresh and/or FET cycle	Biopsy catheter	Introduction of biopsy catheter in endocervical canal	LB	15	38.6 (34–44)	21.3 (16–28)	64.8 (12–204)	1.0 (1–1)

RCT, randomized controlled trial; FET, frozen embryo transfer. LB, live birth. NA, not available.

Overview of all participating studies including information on the publication status, country of origin, study population, and study characteristics.

* The corresponding publication is cited as Polanski *et al*. 2015.

¹ Failed transfers from fresh cycles only.

**Figure 2.**
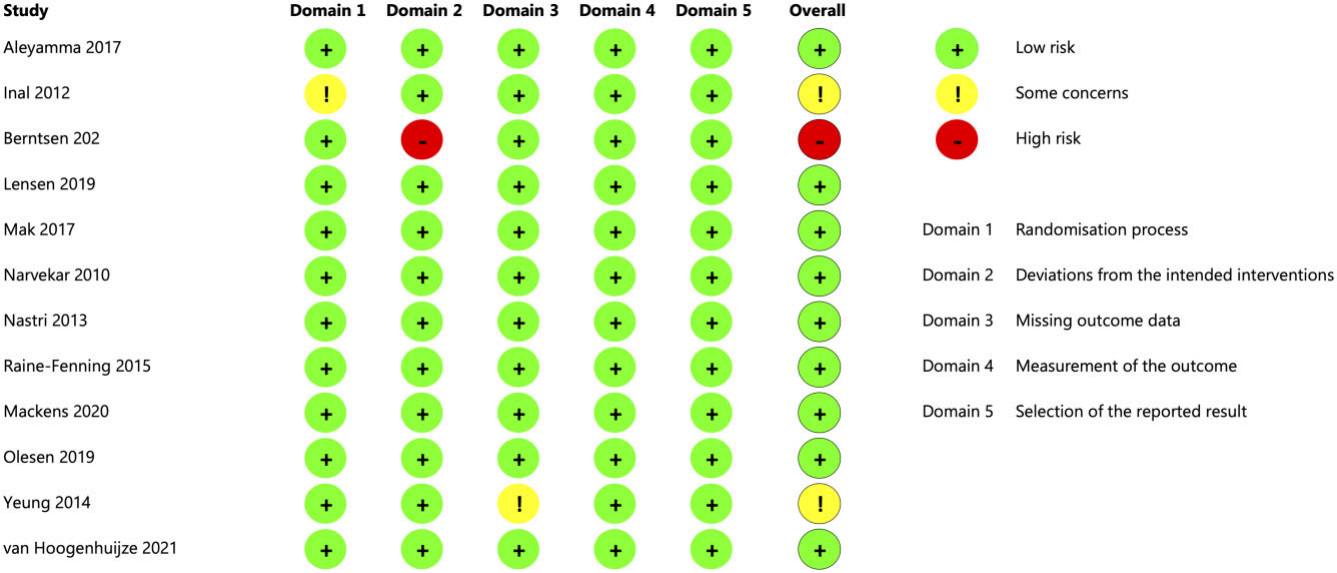
**Risk of bias assessment of the included trials.** Risk of bias assessment according to the Cochrane Risk of Bias Tool 2. All studies that were included in the IPD analysis and had a published manuscript or conference abstract were assessed for risk of bias. Thus, the registered but not published trial by Lee *et al.* was not assessed for risk of bias.

The risk of bias was assessed to be low for 10 studies (Fig. 2). Two were scored with overall ‘some concerns’ due to a lack of information on allocation concealment (İnal, 2012), and a possible relation between missing information and allocation, due to not following-up participants that did not adhere to their allocated treatment (Yeung *et al.*, 2014). One study was scored with a high risk of bias due to the risk of unbalanced groups due to closure of one participating center and exclusion of women based on findings during the study procedure (Berntsen *et al.*, 2020).

### Descriptive analysis of participants and treatment course

Of the 4112 included participants, 2059 were allocated to scratch and 2053 to control. The median participant age at randomization to scratch or control was 34.3 and 34.6 years, respectively, median BMI 23.3 and 23.7 kg/m^2^, and median duration of infertility was 36.0 months in both groups. In both groups, more than 50% of the participants had had no or one previous failed embryo transfer. The cause of infertility was comparable in both groups, with approximately 22–23% suffering from unexplained infertility, 22–23% suffering from male factor, and the remaining 38–40% being diagnosed with other causes (Table 3; for baseline tables according to the AT and AT+ET principle see Supplementary Tables S3 and S4).

**Table 3.** Baseline characteristics of participants according to allocated treatment (intention to treat).

	Scratch (n = 2059)		Control (n = 2053)	
**Female age (years)** ^a^	34.3	(31.0–37.9)	34.6	(31.3–38.0)
**Female BMI (kg/m^2^)** ^b^	23.3	(21.0–26.4)	23.7	(21.3–26.8)
**Duration of infertility (months)** ^c^	36.0	(24.0–58.3)	36.0	(24.0–60.0)
**Number of previous failed embryo transfers** ^d^				
0	564	(27.4%)	564	(27.5%)
1	552	(26.8%)	626	(30.5%)
2	401	(19.5%)	347	(16.9%)
3	194	(9.4%)	190	(9.3%)
≥4	208	(10.1%)	200	(9.7%)
**Smoking status of the female** ^e^				
No	1445	(70.2%)	1447	(70.5%)
Yes	103	(5.0%)	105	(5.1%)
Quit	10	(0.5%)	6	(0.3%)
**Gravidity of the female** ^f^				
0	812	(39.4%)	780	(38.0%)
1	377	(18.3%)	394	(19.2%)
2	147	(7.1%)	146	(7.1%)
≥3	100	(4.9%)	114	(5.6%)
**Parity of the female** ^g^				
0	719	(34.9%)	759	(37.0%)
1	154	(7.5%)	133	(6.5%)
2	19	(0.9%)	14	(0.7%)
≥3	6	(0.3%)	8	(0.4%)
**Primary infertility of the female?** ^h^				
No	658	(32.0%)	685	(33.4%)
Yes	1062	(51.6%)	1038	(50.6%)
**Cause of infertility** ^i^				
Unexplained	477	(23.2%)	454	(22.1%)
Male factor	477	(23.2%)	461	(22.5%)
Other	792	(38.5%)	800	(40.0%)

Observed data only. Data are median (interquartile range) or n (% of randomized population). 13 trials.

Overview of the participant characteristics at randomization. The number of previous failed embryo transfers refers to the number of failed attempts for the current childwish, i.e. excluding embryo transfers for a previous child. For cause of infertility, ‘other’ includes tubal factor, endometriosis (grade I–IV), ovulatory disorder, and other causes.

a Data are missing for: Overall analysis: 10 and 11 participants, resp.

b Data are missing for: Overall analysis: 124 and 133 participants, resp.

c Data are missing for: Overall analysis: 559 and 549 participants, resp.

d Data are missing for: Overall analysis: 140 and 126 participants, resp.

e Data are missing for: Overall analysis: 501 and 495 participants, resp.

f Data are missing for: Overall analysis: 623 and 619 participants, resp.

g Data are missing for: Overall analysis: 1161 and 1139 participants, resp.

h Data are missing for: Overall analysis: 339 and 330 participants, resp.

i Data are missing for: Overall analysis: 328 and 323 participants, resp.

The post-randomization trajectory that participants underwent is summarized in Table 4. The large majority adhered to their allocated treatment, but cross-over occurred slightly more in the allocated scratch group. Other treatment characteristics were comparable in both groups, with the majority undergoing a fresh transfer, and approximately 50% undergoing a single embryo transfer.

**Table 4.** Descriptive analysis of treatment characteristics according to allocated treatment (intention to treat).

	Scratch (n = 2059)		Control (n = 2053)	
**Study treatment received** ^a^				
Scratch	1904	(92.5%)	5	(0.2%)
No intervention/sham procedure	75	(3.6%)	2047	(99.7%)
**1st IVF/ICSI/FET treatment post-randomization** ^b^	*P = 0*.*057*			
Fresh IVF/ICSI	1426	(69.3%)	1373	(66.9%)
FET	263	(12.8%)	238	(11.6%)
Cancel/no embryo for transfer	329	(16.0%)	380	(18.5%)
**Number of embryos transferred** ^c^	*P = 0*.*118*			
No transfer	329	(16.0%)	380	(18.5%)
SET	1101	(53.3%)	1027	(50.0%)
DET	343	(16.7%)	351	(17.1%)
TET	66	(3.2%)	74	(3.6%)
QET or more	23	(1.1%)	18	(0.9%)

Observed data only. Data are n (% of randomized participants). FET, frozen embryo transfer; SET, single embryo transfer; DET, double embryo transfer; TET, triple embryo transfer; QET, quadruple embryo transfer; n.a., not applicable.

Overview of the treatment characteristics of the first planned treatment after randomization. Comparative analysis for the type of treatment and the number of embryos transferred showed no significant differences between the randomized groups.

a Data missing for scratch and control: 80 and 1 participants, resp.

b Data missing for scratch and control: 41 and 62 participants, resp.

c Data missing for scratch and control: 197 and 203 participants, resp.

Pain experienced during the scratch was recorded by four trials (Nastri *et al.*, 2013; Mak *et al.*, 2017; Lensen *et al.*, 2019; van Hoogenhuijze *et al.*, 2020), of which two trials included a sham procedure so that pain was also recorded for the control group (Nastri *et al.*, 2013; Mak *et al.*, 2017). As shown in Table 5, the median experienced pain in the scratch group was 4.0 on a scale of 0–10 (IQR 2.2–6.0). The two studies that recorded pain scores for both the scratch and control group showed that the median pain was higher in the scratch group compared to controls: 4.8 (IQR 3.0–7.0) versus 1.0 (0.3–2.0). These results were not tested for significance as they represent only 2/13 studies with 342/4112 participants.

**Table 5.** Pain and adverse events from the endometrial scratch.

	Scratch (n = 2059)			Control (n = 2053)		
**Pain** ^a^			n			n
Report from all 4 studies	4.0	(2.2–6.0)	1253			
2 studies with sham procedure^b^	4.8	(3.0–7.0)	172	1.0	(0.3–2.0)	170
**Adverse events** ^c^	*Events*					
No adverse events	329					
Bleeding	91					
Abdominal pain	44					
Bleeding and abdominal pain	111					
Other	8					
**Actions taken upon adverse event** ^d^	*Events*		*Total number of adverse events: 254*			
None, expectant without physician’s consult	228					
Consulted physician, expectant management	1					
Other^e^	14					

Pain is reported as a median (interquartile range). n, number of participants. Observed data only.

Pain during the endometrial scratch and adverse events after the endometrial scratch were systematically recorded by four trials, of which two trials performed a sham procedure in the control group. Only the trials performing the sham procedure recorded pain in both groups. None of the trials recorded adverse events in the control group.

a Reported by numeric rating scale (NRS) or visual analogue scale (VAS). Recorded by four trials: Lensen, Nastri, van Hoogenhuijze, and Mak. Two of these trials, Nastri and Mak, performed a sham procedure in the control group, and also recorded pain scores in the control group.

b Sham procedure was ‘drying the cervix with a gauze’ (n = 79) or ‘endocervical manipulation’ (n = 91).

c Reported for a total of 583 out of 1262 randomized participants by four trials: Aleyamma, Inal, Lensen, and van Hoogenhuijze. Only recorded for the scratch group.

d Reported for 243 out of 254 participants with known adverse events. Reported by two trials: Lensen and van Hoogenhuijze. Only recorded for the scratch group.

e For these women, it was known that they were *not* hospitalized, but apart from that it was unknown how their symptoms were handled. Symptoms were after a mid-luteal phase scratch and included ‘minimal bleeding and heavy abdominal pain’ (n = 6), ‘heavy bleeding and minimal abdominal pain’ (n = 1), ‘minimal bleeding and fever’ (n = 1), ‘fever’ (n = 1), ‘heavy abdominal pain’ (n = 3), ‘heavy bleeding’ (n = 2).

The occurrence of adverse events, such as bleeding and abdominal pain, were reported by four trials and for the scratch groups only and are shown in Table 5 (İnal, 2012; Aleyamma *et al.*, 2017; Lensen *et al.*, 2019; van Hoogenhuijze *et al.*, 2020). Out of 583 women, 329 experienced no adverse events, while 111 women reported bleeding and abdominal pain, 91 bleeding only, 44 abdominal pain only, and eight reported other symptoms. Of the 254 women that experienced any adverse event, 228 did not contact a physician, one contacted a physician but did not receive further treatment, and for 25 women it was unknown how their symptoms were handled, although for 14 out of these 25 it was known that they were not hospitalized (Table 5).

### Primary outcome (live birth)

The overall, one-stage ITT analysis for LB showed an OR of 1.27 [95% CI 1.04–1.54] for the unadjusted model, and an OR of 1.29 [95% CI 1.02–1.64] with adjustment for age, BMI, duration of infertility, number of previous failed transfers, cause of infertility, and treatment type (Table 6). The sensitivity analyses based on the risk of bias showed comparable results, where only the one-stage adjusted model for low-risk-of-bias studies was not significant (Table 6).

**Table 6.** Results for the outcome live birth.

Outcome	Studies	Participants	Method	OR	95% CI	95% PI	Intercept	Trtm-effect	Significant adjustments
ITT Overall	13	4112	Two-Stage	1.28	[1.02 , 1.61]*	[0.80 , 2.05]	Fixed	Random	n.a.
			One-Stage unadjusted	1.27	[1.04 , 1.54]*	[0.88 , 1.83]	Random	Random	n.a.
			One-Stage adjusted^a^	1.29	[1.02 , 1.64]*	[0.74 , 2.25]	Random	Random	Age, undergoing fertility treatment
ITT Sensitivity	11	3883	One-Stage unadjusted	1.26	[1.03 , 1.54]*	[0.89 , 1.79]	Random	Random	n.a.
Low & Medium RoB			One-Stage adjusted^a^	1.28	[1.00 , 1.63]*	[0.74 , 2.20]	Random	Random	Age, undergoing fertility treatment
ITT Sensitivity	9	3508	One-Stage unadjusted	1.26	[1.03 , 1.55]*	[0.93 , 1.71]	Random	Random	n.a.
Low RoB			One-Stage adjusted^a^	1.28	[0.99 , 1.65]	[0.78 , 2.10]	Random	Random	Age, undergoing fertility treatment, duration of infertility
AT Overall	12	3954	Two-Stage	1.24	[0.99 , 1.54]	[0.83 , 1.84]	Fixed	Random	n.a.
			One-Stage unadjusted	1.22	[1.01 , 1.47]*	[0.90 , 1.65]	Random	Random	n.a.
			One-Stage adjusted^a^	1.22	[0.96 , 1.54]	[0.73 , 2.02]	Random	Random	Age, undergoing fertility treatment, duration of infertility
AT+ET Overall	12	3250–3257^b^	Two-Stage	1.25	[0.97 , 1.60]	[0.73 , 2.12]	Fixed	Random	n.a.
			One-stage unadjusted	1.24	[0.99 , 1.54]	[0.81 , 1.89]	Random	Random	n.a.
			One-stage adjusted	1.25	[0.99 , 1.57]	[0.77 , 2.03]	Random	Random	Age, duration of infertility
AT+ET Sensitivity	12	3250–3257^b^	One-stage adjusted *sensitivity*^c^	1.24	[0.99 , 1.55]	[0.79 , 1.94]	Random	Random	Age, duration of infertility

* *P* < 0.05.

OR, odds ratio; PI, prediction interval; Trtm effect, treatment effect; n.a., not applicable; RoB, risk of bias; ITT, intention to treat; AT, as treated; AT+ET, as treated analysis of those participants who underwent an embryo transfer.

Results of all analyses for the primary outcome ‘live birth’. The intention to treat analysis was also performed as a sensitivity analysis according to the level of risk of bias, meaning that in the ‘low&medium RoB’ analysis all studies with high RoB were excluded, and the ‘low RoB’ analysis both studies with high and medium RoB were excluded.

a Adjusted for age (grand mean-centered), BMI (grand mean-centered), duration of infertility (grand mean-centered, in months), number of previous failed transfers, cause of infertility (unexplained=reference, male factor, other), and type of treatment (no treatment/embryo transfer=reference, fresh, frozen). Thus, the reference patient has average age, average BMI, average duration of infertility, and no previous failed transfers. Additionally, in adjusted models, the reference patient has a diagnosis of unexplained infertility and has undergone no fertility treatment.

b The number of participants varies between 3250 and 3257 in each of the 45 imputation rounds, because for 57 participants it was imputed whether or not an embryo transfer was performed.

c AT+ET sensitivity analysis: one additional adjustment factor was added to the analysis, namely the number of embryos that were transferred in the study cycle. This was defined as a single embryo transfer (SET), double embryo transfer (DET), or triple or more embryo transfer (TET or more).

The outcome live birth was imputed for: ITT overall 100 participants, ITT low risk&some concerns 94 participants, ITT low risk 91 participants, AT 100 participants, AT+ET 6 participants.

**Figure 3.**
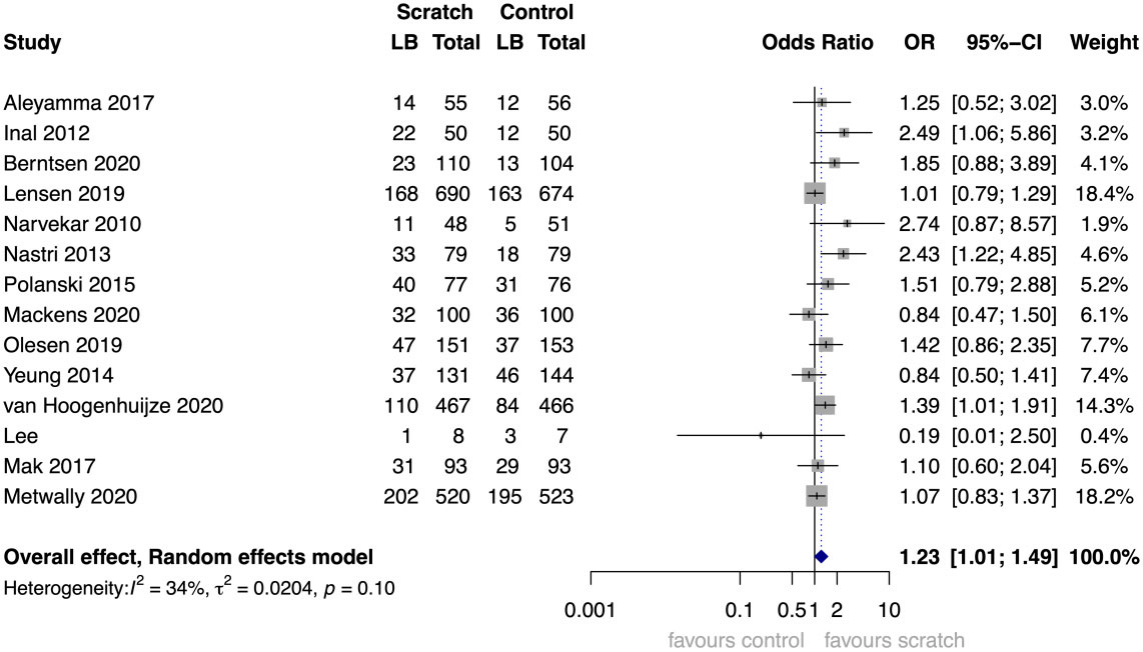
**Forest plot of two-stage Intention to Treat analysis, outcome: live birth.** Analysis of studies that did share IPD combined with a recent, yet unpublished, study that did not (yet) share IPD. OR, odds ratio. 14 studies (5155 participants). Shown event numbers are observed values only. This forest plot shows the two-stage analysis of the 13 studies that were included in the IPD analysis combined with a single recent RCT that has been published while drafting this manuscript but that could not yet share IPD (Metwally *et al.*, 2020). For the 13 studies that shared IPD, the per-study and pooled odds ratios are based on imputed data in our IPD dataset. The outcome ‘live birth’ was imputed for 100 participants (fraction of missing outcome data 2.4%). For the single study that did not share IPD (Metwally *et al.*, 2020), the data were withdrawn from their manuscript. Numbers of missing data are unknown.

The one-stage AT and AT+ET analyses, as well as the two-stage ITT, AT, and AT+ET analyses, yielded similar results as the primary one-stage ITT model, with OR varying between 1.22 and 1.28, and 95% CIs hovering just above and just below the significance level (Table 6, Fig. 3, Supplementary Figs S1 and S2).

### Secondary outcomes

#### Ongoing pregnancy and clinical pregnancy

For the secondary outcomes of OP and CP, the one-stage and two-stage analyses according to the ITT, AT, and AT+ET principles all showed results in the same direction as the outcomes for LB. Odds ratios varied between 1.18 and 1.25 for OP [95% CIs from 0.94–1.49 up to 1.01–1.50] and between 1.20 and 1.30 for CP [95% CIs from 0.96–1.51 to 1.04–1.40] (Supplementary Tables S5, S6, and S7; forest plots of two-stage analyses in Supplementary Figs S3, S4, S5, S6, S7, and S8).

#### Miscarriage rate

The miscarriage rate was analyzed as the number of miscarriages compared to the number of clinical pregnancies and was comparable between the scratch and control groups in the ITT, AT, and AT+ET analyses, as well as for the two-stage and one-stage analyses. Odds ratios varied between 0.96 and 1.01 for the different types of analyses, with 95% CIs all covering a wide range around 1 (Supplementary Tables S5, S6, and S7; forest plots of two-stage analyses in Supplementary Figs S9, S10, and S11).

#### Ongoing multiple pregnancy

No formal comparative analysis was performed for ongoing multiple pregnancy (i.e. more than one live fetus on ultrasound at 10 weeks’ gestational age) due to the low number of events. A total of 98 out of 4112 women were reported to have an ongoing multiple pregnancy, of whom 45 were allocated to control and 53 to scratch. Importantly, data were missing for 573 participants (Supplementary Table S8).

#### Ectopic pregnancy

Similarly, due to a low rate of occurrence, no comparative analysis was performed for the outcome ectopic pregnancy. An ectopic pregnancy was diagnosed in seven women in control, and eight women in the scratch group, with data missing for 468 out of 4112 women (Supplementary Table S8).

#### Treatment–covariate interaction

**Figure 4.**
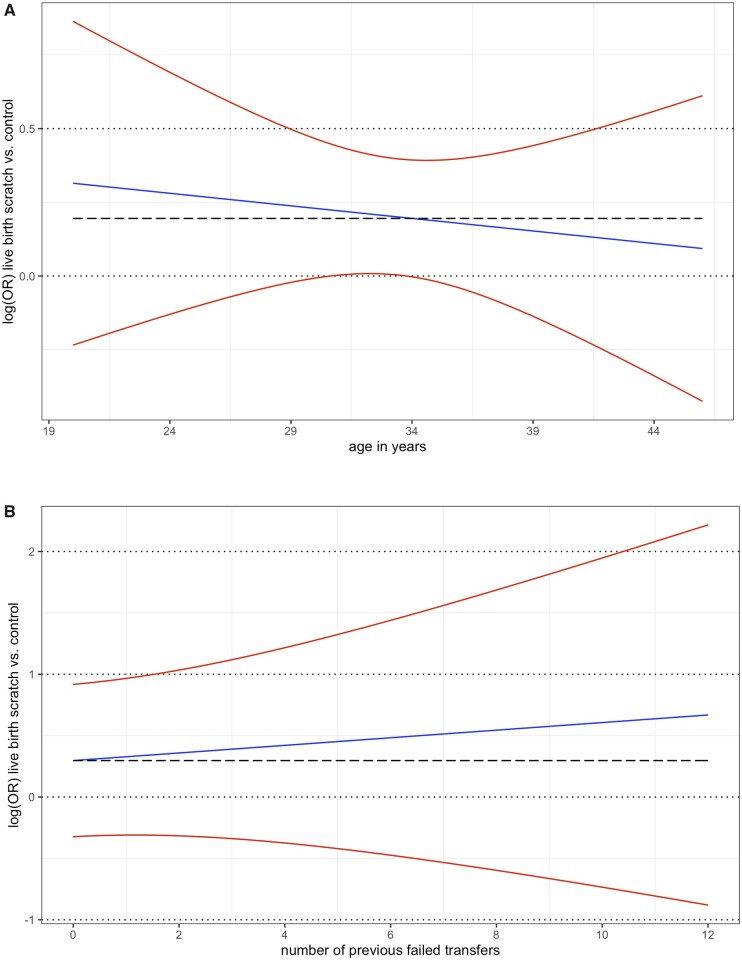
**Participant-level interaction of age and previous failed transfers with the scratch-effect on live birth.** (**A**) Plot of the interaction between participant age and the scratch-effect on live birth at participant level (one-stage within-trial analysis). The log-OR of scratch versus control for live birth is represented as a function of age (blue line), with pointwise 95% CI (red lines). The slope of the blue line is −0.010 (standard error (SE) 0.02). The black striped line indicates the log-OR of the reference participant with a mean age. *P*-value 0.60. (**B**) Plot of the interaction between the number of previous failed embryo transfers (participant-level) and the scratch-effect on live birth (one-stage within-trial analysis). The log-OR of scratch versus control for live birth is represented as a function of previous failed embryo transfers (blue line), with pointwise 95% CI (red lines). The slope of the blue line is 0.04 (SE 0.06). The black striped line indicates the log-OR of the reference participant with 0 previous failed embryo transfers. *P*-value 0.23.

Within-trial analysis revealed that none of the studied participant characteristics interacted with the scratch effect on the chance of LB (Table 7). Thus, the number of previous failed transfers, participant age, cause of infertility (according to our categorization), and treatment type (fresh versus frozen embryo transfer) were not found to modify the effect of endometrial scratching on the chance of a LB. For the continuous parameters of participant age and number of previous failed embryo transfers, the logOR with 95% CI is shown for each age and for each number of previous failed transfers in Fig. 4.

**Table 7.** Interactions of patient characteristics with scratch effect on live birth.

	As treated with embryo transfer
Hypothesized effect modifier	*P*-value
**Previous failed transfers**	
Within trial (deviation from study mean)	0.517
Across trial (study means)	0.809
**Participant age**	
Within trial (deviation from study mean)	0.601
Across trial (study means)	0.032*
**Infertility cause** *within trial only*	
Unexplained versus Male factor versus Other^a^	0.704
**Treatment type** *within trial only*	
Fresh versus Frozen embryo transfer	0.334

Analysis to define whether the effect of endometrial scratching on live birth changes across varying patient characteristics, assessed using interaction analysis. Calculations are based on the as treated with embryo transfer (AT+ET) model for the outcome live birth, and on imputed data. 11 studies, 3241 participants. Data were imputed for age (n = 21), previous failed transfers (n = 266), infertility cause (n = 651), treatment type (n = 103). Interactions were tested in a one-stage model. The within-trial analysis is most important in evaluating the variation of treatment effect across participant characteristics. The study by Lee was excluded due to its small sample size (n = 15), which precludes analysis of within-study interactions.

a *P*-value calculated using the likelihood ratio test, comparing two generalized linear mixed regression models with and without the interaction for infertility cause.

* significant *p*-value at 0.05 level.

**Figure 5.**
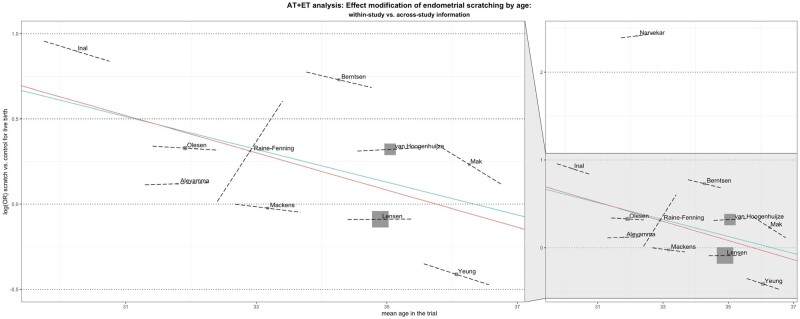
**Interaction of age with scratch-effect on live birth, combined representation of IPD participant-level analysis, IPD study-level analysis, and analysis within each trial.** Plot of the interaction between age and the scratch-effect on live birth, at participant- and study-level. The red line represents the log-OR of scratch versus control for live birth as a function of *mean participant age* (one-stage participant-level analysis), and the green line represents the meta-regression of per-trial treatment effect estimates versus mean participant age (two-stage study-level analysis). The dashed black lines represent the interaction between age and treatment effect on live birth *within* each trial (two-stage analysis).

The only significant interaction was at study-level for the mean participant age per study. Studies with a younger mean age showed on average larger effects of endometrial scratching on live birth (*P* = 0.032). As we did not find such a relation on participant-level, we further evaluated whether this finding could be a true participant-level interaction or whether it was caused by aggregation bias, which is the wrongful assumption that a trend in pooled results also applies to individual participants. We conducted a *post-hoc* two-stage meta-analysis in which we first fitted a multivariate logistic regression model including the interaction with age within each study, and then pooled the estimates of the interaction effect in a random-effects model and restricted maximum likelihood estimation. We then plotted in one figure the one-stage between-trial interaction, the meta-regression of per-trial treatment effect estimates versus the mean participant age, and each study’s within-trial interaction (Fig. 5). Most within-trial interaction graphs do not show that a younger age is related to a larger effect, and therefore we interpreted the significant interaction for the mean participant age (study-level interaction) as being an effect of aggregation bias.

#### Scratch timing

The fifth interaction, scratch timing, was impossible to study at participant-level due to a lack of data. The timing of the scratch, however, has been suggested as a possible cause for the variation in observed treatment effects in the various RCTs (Odendaal and Quenby, 2019). We therefore opted for a two-stage meta-analysis in which studies were grouped according to scratch timing as follows: a single or double scratch in the luteal phase of a natural, pre-transfer cycle versus a single scratch in an oral contraceptive cycle timed 5–14 days prior to the start of ovarian stimulation versus any other timing in natural or oral contraceptive cycles. This is basically a study-level analysis, except that the participants from the RCT by van Hoogenhuijze were categorized in the first and second category (van Hoogenhuijze *et al.*, 2020). This showed that the nine studies that conducted a single or double scratch in the luteal phase of a natural cycle, preceding the embryo transfer cycle, yielded a pooled OR of 1.27 [95% CI 0.96–1.68] (Supplementary Fig. S12). The two studies that performed the scratch 5–14 days prior to the start of ovarian stimulation in an oral contraceptive cycle yielded an OR of 1.00 [95% CI 0.01–173.75]. Studies with other or mixed timing in mixed natural and oral contraceptive cycles showed an overall OR of 1.19 [95% CI 0.59–2.42]. Owing to the lower number of included studies and participants per category, uncertainty is relatively high—especially in the oral contraceptive and the mixed timing groups. This is also reflected by the wide CIs.

### Publication and IPD availability bias

**Figure 6.**
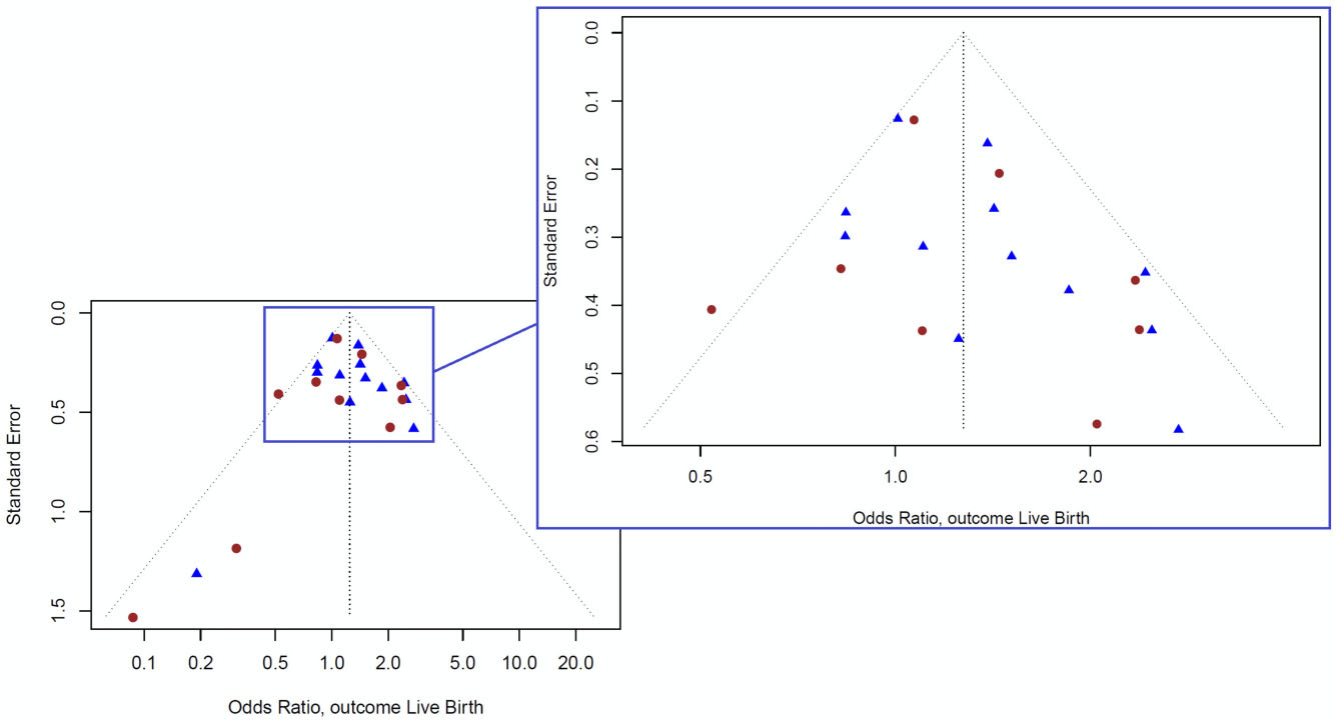
**Funnel plot of studies that did and did not share IPD and reported live birth.** Two-stage ITT analysis, outcome live birth. Blue triangles 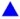: studies that did share IPD. Red dots 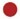: studies that did not share IPD. Funnel plot representing all published trials and their reported endometrial scratching effect on live birth. The funnel (black dotted lines) indicates the lower and upper limits of the 95% confidence region. The black dotted mid-line is the pooled effect (OR) of endometrial scratching on live birth. The slight asymmetry to the left corner could indicate either publication bias or small study effect. IPD, individual participant data; *Y*-axis, standard error; *X*-axis, scratching effect on live birth, expressed as OR.

The funnel plot of participating (IPD) studies and non-participating (AD) studies shows that all but one study fall within the expected 95% range (Fig. 6). The funnel is slightly asymmetric with a few outliers in the lower left corner, which could represent either publication bias or small study effect. No evident difference is observed between the IPD and AD studies.

**Figure 7.**
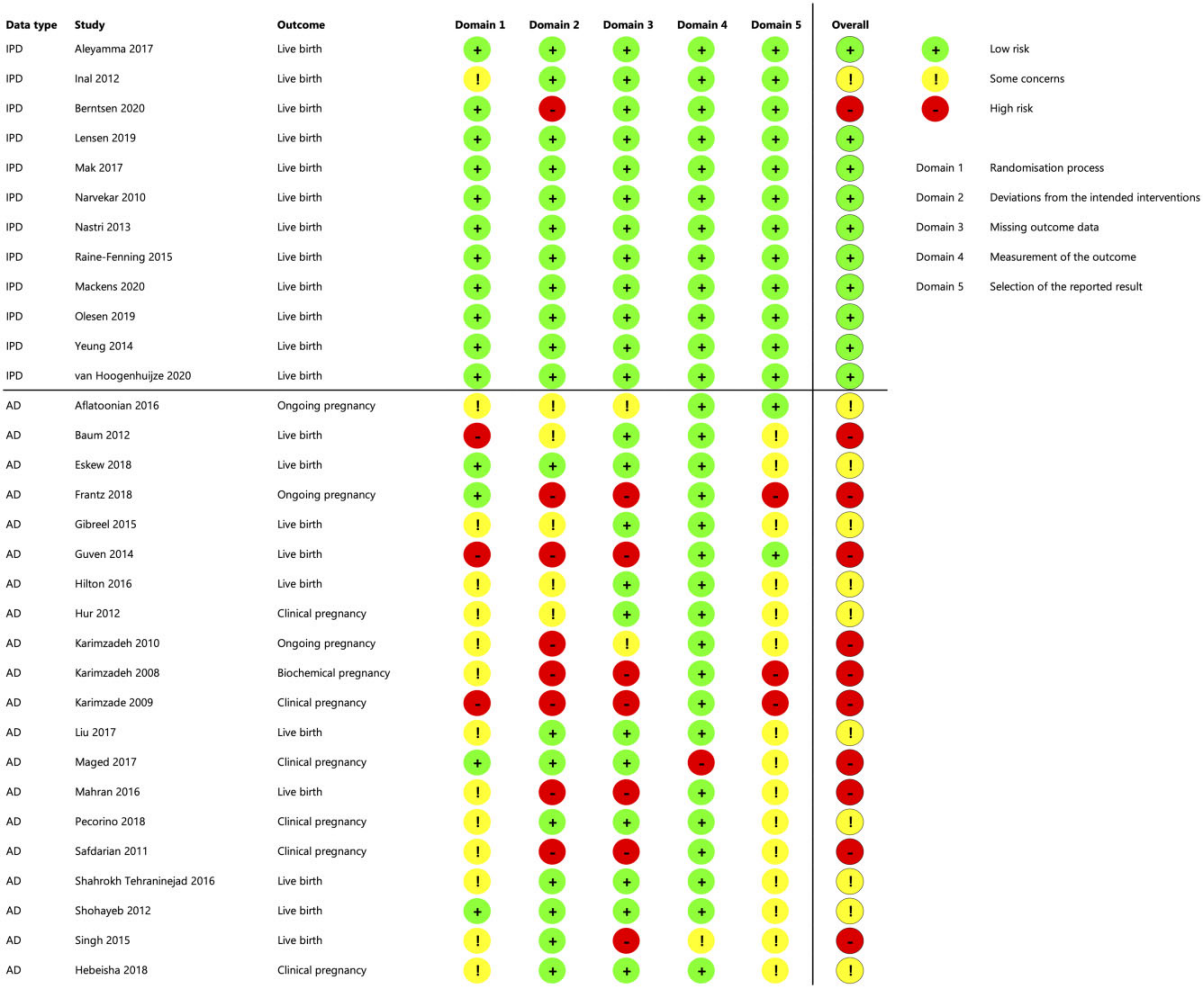
**Risk of bias of studies that did and did not share IPD.** The Cochrane Risk of Bias Tool 2 was used. Overview of the Risk of Bias assessment of all published studies. Studies that were registered but unpublished could not be assessed. Studies above the dashed line were included in the IPD analysis. Studies below the dashed line did not share IPD, or were excluded from IPD analysis (Mahran *et al.*, 2016; Hebeisha *et al.*, 2018). IPD, individual participant data available; AD, only aggregate data available.

Risk of bias assessment of published studies showed that non-participating studies were considered to yield a higher risk of bias (Fig. 7). This frequently stemmed from unclear randomization processes (e.g. generation of the randomization sequence and/or allocation concealment, domain 1), a high number of participants that did not adhere to the allocated treatment or additional in-/exclusion criteria that could only be assessed *after* randomization (e.g. requirement of a high quality embryo for transfer, domain 2), or not reporting according to ITT principles by excluding women who conceived naturally or did not reach embryo transfer (domain 5). Importantly, the IPD studies were assessed based on all information available, including the published manuscripts but also study protocols and received data, whereas the AD studies were assessed based on published manuscripts only.

**Figure 8.**
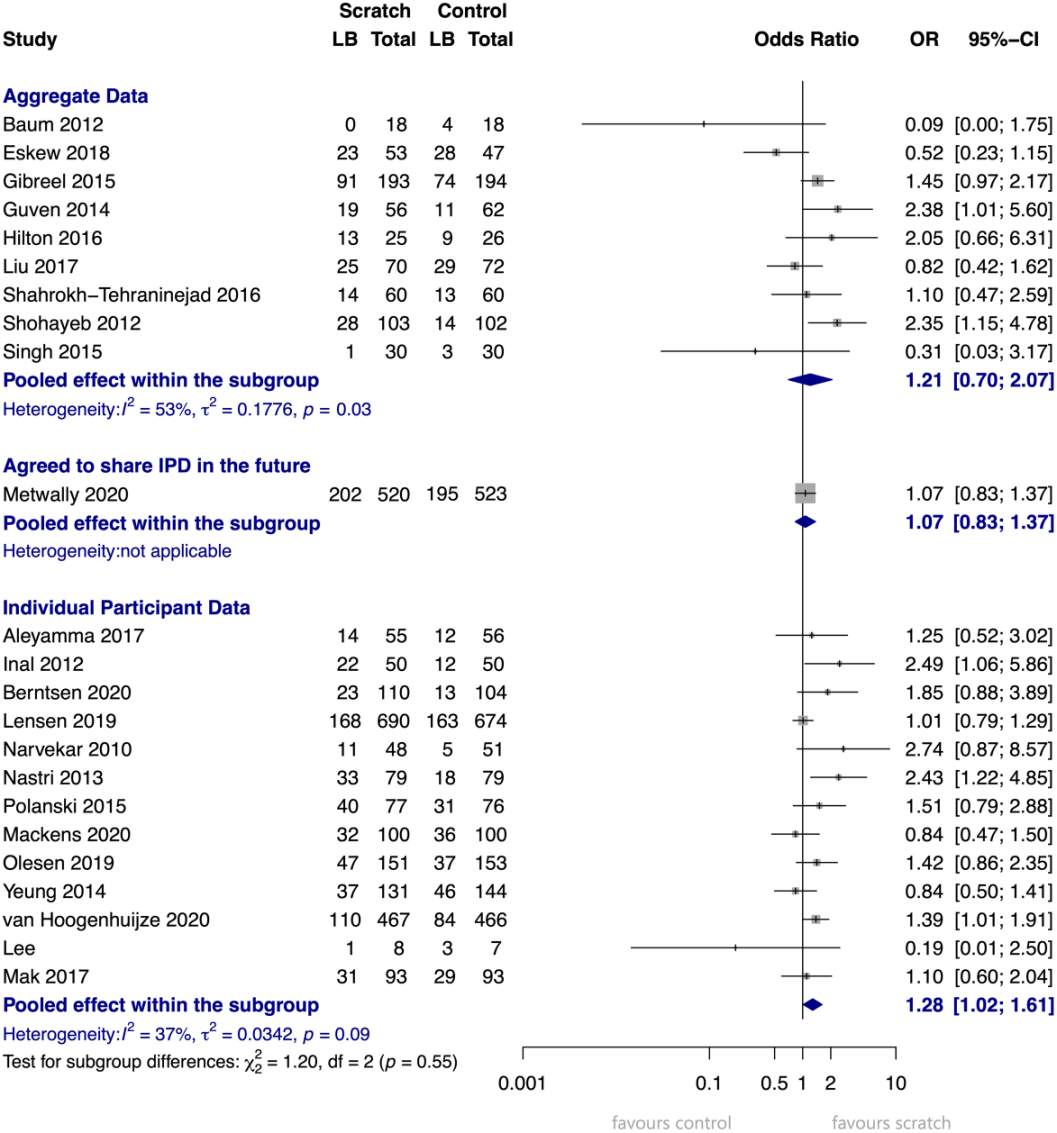
**Forest plots of studies that did and did not share IPD.** Two-stage intention to treat analysis, outcome live birth. 23 studies representing 6374 participants: 10 with aggregate data representing 2262 participants and 13 with IPD representing 4112 participants. Forest plots of the two-stage intention to treat analysis of the outcome live birth, split into three analyses: studies that shared IPD and were included in the IPD analysis, studies that were not yet able to share data, and studies that declined participation in the IPD or could not be contacted despite multiple attempts. Aggregate data events, total numbers, and calculations are based on the numbers as reported in the manuscript, where we have tried to adhere to the intention to treat principle as much as possible. Individual participant data events and total numbers are based on observed values only, representing the individual participants that were included in the final analysis of this IPD-MA. The calculations for OR and 95% CI are based on the imputed data included in this IPD-MA. LB, live birth; OR, odds ratio. Aggregate data: studies that did not share individual participant data (IPD).

A conventional, two-stage meta-analysis was performed to compare whether the pooled outcome of AD studies differs from the IPD studies. In Fig. 8, the pooled OR of the AD studies (OR 1.21) is in line with that of the IPD studies (OR 1.28), but the 95% CI of the AD studies is much wider and includes the ‘1’. The level of heterogeneity, assessed using the I^2^ statistic, is also higher in the AD studies compared to IPD studies, namely 53% versus 37%. The study by Metwally is categorized separately, as they did agree to participate but were unable to share their data yet (Metwally *et al.*, 2020). Taken altogether, this forest plot does not show that the non-participating studies found systematically more negative or more positive results than the studies that participated.

## Discussion

In this IPD meta-analysis on endometrial scratching in women undergoing IVF/ICSI we included data of 4112 participants from 13 studies. A one-stage ITT analysis showed increased odds for a LB after endometrial scratching. Additional analyses based on AT and AT+ET were in line with these results but could not rule out the possibilities of none or a small (negative) treatment effect. Subgroup analysis did not find evidence that participant age, number of previous failed transfers, cause of infertility or fresh versus frozen embryo transfer modified the effect of endometrial scratching.

### Relation to current available literature

While the earliest RCTs on endometrial scratching showed (strong) significant effects of scratching (Shohayeb and El-Khayat, 2012; Guven *et al.*, 2014), the more recent publications showed either no effect (Lensen *et al.*, 2019) or non-significant positive effects (Olesen *et al.*, 2019; Berntsen *et al.*, 2020), while some even observed a possible harmful effect due to an increased miscarriage rate (Mackens *et al.*, 2020). The wide variation in results, combined with varying definitions of pregnancy outcomes, complicated interpretation of the ‘true’ scratch effect. Several conventional meta-analyses were conducted, all without being able to compensate for the lack of uniformity in definitions and all drawing different conclusions (Panagiotopoulou *et al.*, 2015; Santamaria *et al.*, 2016; van Hoogenhuijze *et al.*, 2019; Lensen *et al.*, 2021).

This IPD-MA addresses these problems in various ways: it allowed for increased homogeneity in the inclusion of participants (e.g. some participants of whom data were available were excluded by the original studies while they would have been included in other studies), it filtered out studies with data integrity concerns by comparing the received IPD with the original published results, it motivated researchers to look up additional data (e.g. on LB), it allowed for increased uniformity in outcome definitions, and it enabled a more precise estimation of the true treatment effect because of the possibility to adjust for participant characteristics as well as true interaction analysis. Another strength is that the risk of publication bias was reduced by including both published and unpublished data, while limiting small study effect by using a weighted model.

The overall results of this IPD-MA are in line with previous conventional meta-analyses; the most recent being the Cochrane update, which provides a somewhat lower estimated OR and a narrower CI but nevertheless is in congruence with the findings of this IPD-MA (Lensen *et al.*, 2021). Furthermore, this IPD-MA provides more confidence in the quality of the data and, most importantly, provides insight into possible subgroup effects, as assessed by interaction analysis. Furthermore, maximal effort was taken to reduce the risk of publication bias by also inviting registered but unpublished trials to share data.

These advantages of IPD-MA are also reflected in our risk of bias assessment, which is overall more optimistic than those in conventional meta-analyses. The fact that in IPD-MA the raw data are used instead as part of a much larger dataset takes away some risks of biases that apply to individual studies or pooled data. This is reflected by the development of a Cochrane Risk of Bias Tool 2, which assesses different domains than Tool 1 used for conventional meta-analyses (Cochrane, n.d.). Also, having available much more information—raw data, study protocol, additional unpublished data, close contact with authors—resolved many issues and doubts.

A possible limitation of this IPD-MA is that not all available studies participated. To cope with the fact that a large, yet unpublished study (Metwally *et al.*, 2020) could not yet share their data, we included their aggregate data in the two-stage analysis, which showed it to be consistent with the one-stage results on the current IPD-only data. Also, risk of bias evaluation revealed that the participating studies were in general rated higher than the non-participating studies. Reasons for not sharing data may be a spectrum from true unavailability, governance problems, low quality of studies, and data integrity issues—we are thus left in the unknown on how big a loss it is that not all studies participated. Be that as it may, as this IPD-MA included the largest and most recent studies, we think that it is representative of the complete studied population and also represents current-day practice on different continents.

Another limitation is that the effect of the timing of the scratching procedure, and whether it was performed in a natural or hormonal contraceptive cycle, could not be evaluated on participant-level. The surrogate two-stage analysis could be optimized compared to previous conventional meta-analyses because the data by van Hoogenhuijze *et al.* could be split across two groups, but apart from that it remains a study-level analysis. Thus, this analysis should not be used for advice at an individual level of what the optimal timing of a scratch should be. Instead, it should be regarded as a stepping stone from which future research can take off. Furthermore, adjustment for embryo quality and embryo stage was not possible because of limited information and varying standards to report embryo quality.

### Implications for future research

Many RCTs and meta-analyses have been carried out thus far, and this IPD-MA adds to that list. All results point in the direction that endometrial scratching may improve LBRs with in the worst case no or a small negative effect (given the 95% CIs of around 1). While the search for the ‘true effect’ may continue by gathering even more data—the easiest way would be to share IPD of all studies that already have been conducted—we also think that there should be a shift of focus to evaluating the technique and timing of scratching alongside its possible mechanism. The two-stage scratch-timing analysis in this IPD-MA suggest that luteal phase, natural cycle scratching may be superior to other timed and/or contraceptive cycle scratching. While this analysis is too imprecise to draw conclusions from, it could be used for generating hypotheses for future research. Furthermore, this IPD-MA illustrated once more the importance of using standardized outcomes and definitions in fertility research, so that outcomes can be easily compared and possible modifying factors, such as embryo quality, can be taken into account.

Future studies could use the data provided in this IPD as a stepping stone for designing studies to further evaluate whether the method and timing of the scratching procedure affects the chance of LB after endometrial scratching.

### Implications for clinical practice

The results of this IPD-MA suggest that endometrial scratching improves LBRs, although some of the 95% CIs also include the possibility of no or even a small negative effect. How the OR translates to clinical practice depends on the ‘basal’ chance of LB—i.e. without endometrial scratching. Also, we must keep in mind that the OR is adjusted for several factors and thus represents the chances for women with the average age, BMI, number of previous transfers etcetera. As an example, the OR of 1.29 [95% CI 1.02–1.64] (ITT analysis) indicates that if the baseline chance (i.e. *without* scratching) of a LB were 20.0%, the risk rate (RR) would be 1.22 [95% CI 1.02–1.45], and thus translate to a chance of LB of 24.4% [95% CI 20.4–29.0%] *with* endometrial scratching. Likewise, a 25% ‘baseline’ chance of LB (i.e. *without* scratching) corresponds with a RR of 1.20 [95% CI 1.02–1.41] and an estimated chance of LB of 30.1% [95% CI 25.5–36.3%] *with* endometrial scratching.

The hypothesis that specifically women with repeated implantation failure would benefit from scratching could not be confirmed (Barash *et al.*, 2003; Potdar *et al.*, 2012; Nastri *et al.*, 2015). From a pragmatic point of view, one could argue that a single endometrial scratch is associated with relatively low costs and yields low risks for the woman—at least in the short term—and that therefore scratching could be offered to women undergoing IVF/ICSI after being counseled on the uncertainties. On the other hand, a lot about scratching is still unclear, such as repeated scratching (of which it is imaginable that this would be done when scratching was implemented in daily practice), its mechanism of action, and whether timing plays a role. The exact technique of applying the scratch is also not uniform, but most studies used an endometrial biopsy catheter. Some trials also performed a hysteroscopy, of which it could be hypothesized that this may alter the effect size. However, two large, high-quality RCTs on the effect of hysteroscopy before IVF/ICSI found no effect on LBRs (El-Toukhy *et al.*, 2016; Smit *et al.*, 2016). Also, the question of whether scratching is associated with pregnancy complications mediated through altered placentation—such as pregnancy-induced hypertension, pre-eclampsia, or small for gestational age—has also not been answered. As these types of complication could have consequences for both the mother and the child (Woods *et al.*, 2017, 2018), this should be evaluated more intensively. In our current IPD dataset, only two studies recorded information on pregnancy complications so that we were unable to perform IPD analysis on this topic (Chapter 6 in van Hoogenhuijze, 2022; Lensen *et al.*, 2019). Lastly, it has been poorly studied how endometrial scratching impacted the women undergoing it: how did they experience post-procedural pain, how did it affect their overall experience of the treatment, and would they be willing to undergo it repeatedly—if it were beneficial? For these reasons, the use of endometrial scratching in clinical practice should be considered with caution, meaning that patients should be properly counseled on the level of evidence and the uncertainties. Meanwhile, further research, with focus on the timing of scratching, its mechanism of action, and its relation to pregnancy complications, should continue.

## Conclusion

This IPD shows that for women undergoing IVF/ICSI with autologous oocytes, endometrial scratching may improve their chance of a LB. Furthermore, characteristics such as age, the number of previous failed transfers, infertility cause, and treatment type were not found to modify the effect of endometrial scratching. The effect of timing and method of the scratching procedure could not be evaluated at the participant level owing to too little within-study variability, and insufficient data.

## Supplementary Material

dmad014_Supplementary_Data

## Data Availability

The data underlying this article cannot be shared publicly because the data were provided by the collaborating authors under the specific condition that it would be used for this project only. The data may be shared on reasonable request to the corresponding author, and only after consent of each collaborating author of the current project.
