# Supplementary material for: Endometrial scratching in women undergoing IVF/ICSI: an individual participant data meta-analysis
Source: Hum Reprod Update. 2023 Jun 19;29(6):721–40. doi: 10.1093/humupd/dmad014 (PMC10628489; doi:10.1093/humupd/dmad014)
Supplement: dmad014_Supplementary_Data [file dmad014_supplementary_data.zip › dmad014_Supplementary_Data/Supplementary Data File S1.doc]

Supplementary Data File S1

Statistical Analysis Plan IPD-SCRaTCH

G. Lahoz Casarramona, N.E. van Hoogenhuijze, R. Wang, H.L. Torrance, F.J.M. Broekmans, M.J.C. Eijkemans

CONTENT

CONTENT	1

STUDY INFORMATION	5

Study background	5

Design and setting	5

Aim/Objective	5

Study characteristics	6

Study identification and selection	6

Study population and sample	6

Intervention	6

Comparator	7

Outcomes	7

Data collection	7

Software	7

STATISTICAL ANALYSIS I: OVERVIEW	8

Risk of bias, quality appraisal and data checking	8

Risk of bias	8

Quality of the data	8

Overall approach to IPD meta-analysis	9

Type of analysis	9

Protocol violations, withdrawals, missing data, outliers and unexpected irregularities	9

Criteria	9

Methods for imputation of missing data	11

Baseline characteristics	12

Methods	12

Missing values	12

Variables	12

Treatment outcomes	14

Primary	14

Secondary	14

Effect modifiers (subgroup analyses)	16

STATISTICAL ANALYSIS II: PRELIMINARY TWO-STAGE META-ANALYSIS	17

Step 1	17

Step 2	17

STATISTICAL ANALYSIS III: PRIMARY ONE-STAGE META-ANALYSIS	17

STATISTICAL ANALYSIS IV: DESCRIPTIVE ANALYSIS INTERVENTION GROUP	18

Outcome: Pain	18

Outcome: Adverse events	18

EXAMPLE TABLES & FIGURES	19

Table 1. Design features and demographic characteristics of included RCTs	19

Table 2. Overall baseline characteristics	19

Table 3. Descriptive analysis	20

Table 4. Treatment effects on primary and secondary pregnancy outcomes: results of one-stage model (1)	21

Table 5. Effect modifiers (subgroup analyses): results of one-stage model (2)	22

Table 6. Pain and adverse events in the week after scratch (scratch group only)	23

Figure 1. PRISMA-IPD flow diagram	24

Figure 2. Risk of bias of eligible studies	25

Figure 3. Funnel plot for the primary outcome (LBR)	26

Figure 4. Forest plot for primary outcome: live birth rate	27

Figure 5. Forest plot for cumulative LBR	27

Figure 6. Forest plot for OPR	27

Figure 7. Forest plot for CPR	27

Figure 8. Forest plot for miscarriage	27

Figure 9. forest plot for multiple pregnancy	27

Figure 10. Forest plot for ectopic pregnancy	27

REFERENCES	28

**PROTOCOL SIGNATURE SHEET**

| **First agreement on SAP – Version 1.3** | |
| --- | --- |
| **Name** | **Signature** |
| *Principal investigator*  Dr. H.L. Torrance, MD PhD, associate professor Reproductive Medicine | Date: 13 January 2020    Signature: |
| *Investigator*  Drs. N.E. van Hoogenhuijze, MD | Date: 13 January 2020  Signature: |

| **Agreement on minor edits on SAP – Version 1.5** | |
| --- | --- |
| **Name** | **Signature** |
| *Principal investigator*  Dr. F.J.M. Broekmans, MD PhD, professor Reproductive Medicine | Date: 12 April 2021  Signature:   |
| *Investigator*  Drs. N.E. van Hoogenhuijze, MD | Date: 12 April 2021    Signature: |

STUDY INFORMATION

Study background

The success of assisted reproductive technologies (ART) is limited by the low rates of embryo implantation (De Geyte*r et a*l., 2018). Endometrial ‘scratching’—a procedure involving intentional injury to the endometrium with the aim of increasing its receptivity (Nastr*i et a*l., 2015)—has been proposed as a technique holding great promise for the improvement of embryo implantation rates. This controversial intervention has been the object of numerous studies and seen a rapid incorporation into clinical practice, but the evidence on its efficacy remains inconclusive to date. Due to the heterogeneity of the primary studies, systematic reviews and traditional meta-analyses based on aggregate data (AD) have not sufficiently clarified whether endometrial scratching is at all beneficial and, if so, under which circumstances or for which patients (Nastr*i et a*l., 2015; Panagiotopoulou, Karavolos and Choudhary, 2015; Ca*o et a*l., 2018; Vitaglian*o et a*l., 2018, 2019; Gu*i et a*l., 2019; van Hoogenhuijz*e et a*l., 2019).

An individual participant data meta-analysis (IPD-MA) has multiple important advantages over the AD approach, including the possibility to standardize participant inclusion and exclusion criteria, outcomes of interest and methods of analysis across studies, while providing greater power to assess interactions between the effects of treatment and participant-level characteristics (Riley, Lambert and Abo-Zaid, 2010; Tierne*y et a*l., 2015).

Therefore we will perform an IPD-MA to evaluate the effectiveness of endometrial scratching in improving live birth rates in women undergoing ART. Additionally, we will assess whether this effectiveness depends on the type of treatment (fresh IVF or ICSI vs. frozen IVF or ICSI embryo transfer (FET)), the number of previously unsuccessful embryo transfers, or the timing of the endometrial scratch (follicular phase of the cycle prior to ART vs. luteal phase of the cycle prior to ART vs. follicular phase of the ART cycle).

Design and setting

The IPD-SCRaTCH is an international collaborative individual participant data meta-analysis of randomized control trials (RCTs) that study endometrial scratching in women undergoing IVF/ICSI with fresh or frozen embryo transfers.

Aim/Objective

The primary objective is to determine whether endometrial scratching is effective at improving live birth rates in women undergoing fresh/frozen transfer, as compared to no scratching or sham.

Secondary objectives are to study differences in the effect of endometrial scratching (expressed in live birth rate) in women:

1. Undergoing fresh IVF/ICSI transfer or FET
2. At varying stages of the fertility treatment trajectory
3. Undergoing endometrial scratching with different time intervals between scratching and embryo transfer

Study characteristics

Study identification and selection

The studies eligible for the IPD-MA have been identified by two review authors through an extensive search of the Cochrane Central Register of Controlled Trials (CENTRAL), the Cochrane Gynaecology and Fertility Group database, MEDLINE, EMBASE, ClinicalTrials.gov and WHO portal for published or registered randomized controlled trials. Studies will be included if:

- they are randomized controlled trials that compare endometrial scratching to placebo or to no intervention;
- they report the outcomes of interest (see Outcomes below);
- they are completed and with data available before September 2020;
- they report having acquired ethics approval, have shown documentation of ethics approval, or were registered prospectively in a registration database.

Pseudo-randomized trials and other study designs will be excluded.

Post-hoc addition:

- if a trial has agreed to collaborate, but is unable to share the IPD before September 2020, we will perform an additional sensitivity analysis in which the aggregate data of this trial has been added to the IPD of the other collaborating trials. This will be done in a two-stage approach.

Study population and sample

The study population consists of women undergoing IVF/ICSI treatment with own oocytes (both fresh and frozen embryo transfers can be included). The sample consists of all randomized participants for whom IPD is made available by the authors of the included studies. We will not specify any additional participant inclusion or exclusion criteria.

Intervention

Endometrial scratching, defined as intentional injury to the endometrium with the purpose of improving endometrial receptivity.

Endometrial scratching may be performed with a biopsy catheter (e.g. Pipelle de Cornier) or a curette. Procedures that are also likely to cause some endometrial disruption but which are not intentional injury (e.g. hysteroscopy, hysterosalpingogram, saline-infusion ultrasound, etc.), or procedures of which the extent of the endometrial disruption is not visible (e.g. saline infusion), will not be considered scratching, and studies that use these procedures as scratching will not be included.

Comparator

No endometrial scratching or a non-disruptive sham procedure before fresh or frozen transfer.

Studies in which all participants underwent hysteroscopy during the workup will be included. Studies that used hysteroscopy as sham will be excluded.

Outcomes

Studies will be eligible if they report at least one of the following outcomes:

- live birth, defined as birth of a live fetus beyond 24 weeks of gestation after the cycle following randomisation;
- ongoing pregnancy, defined as a positive fetal heartbeat on ultrasound from a gestational age of 10 weeks onwards, after the cycle following randomisation;
- clinical pregnancy, defined as the presence of a gestational sac on ultrasound at a gestational age of 6-7 weeks, after the cycle following randomisation.

Data collection

The authors of the eligible studies will be approached and invited to participate in the IPD-MA. We will provide the necessary documents, mechanisms and resources for data request, coding, transfer and central collection. Each research group will retain ownership of their own data. Coding of the data will guarantee that the key to link IPD to patient identity will remain in the possession of each research group.

For those studies for which IPD cannot be obtained, we will seek to collect the published aggregate data and the reasons for non-availability of IPD.

Software

SPSS (version 25.0) and R will be used for all statistical analyses.

STATISTICAL ANALYSIS I: OVERVIEW

Risk of bias, quality appraisal and data checking

Risk of bias

We will construct the PRISMA-IPD flow diagram (Stewar*t et a*l., 2015) (Figure 1) detailing the process of study selection and collection of IPD and aggregate data. This diagram will report the numbers of studies identified, screened for eligibility, and included or excluded, together with the numbers of participants of these studies and the reasons for exclusion at each stage. The flow diagram will also report the numbers of studies that provided IPD and of those that did not, along with the reasons why IPD could not be obtained in the latter case, and the numbers of participants for which IPD could or could not be obtained.

Two review authors will independently assess study quality and risk of bias. If there are disagreements, a third author will be consulted. The risk of bias will be assessed using Version 2 of the Cochrane risk-of-bias tool for randomized trials (RoB 2). (Stern*e et a*l., 2019) We will assess whether there are any differences in risk of bias between the studies for which IPD could be obtained and the studies for which IPD could not be obtained. Additionally, we will test for publication bias and data availability bias by constructing funnel plots based on the available aggregate data or, where applicable, on the summary effect estimates calculated from IPD.

Post-hoc additional analysis:

After the deadline for obtaining the data had been reached, it became apparent that a relatively large proportion of trials did not share data. This was due to non-responding, declining, or non-responding after initial agreement. In order to evaluate if ‘participation bias’ was present, i.e. if the collaborating studies systematically have different findings from the studies that did not collaborate, Therefore, a two-stage analysis was performed on the studies that did not collaborate and on the studies that did collaborate. A forest plot showing the pooled odds ratios of the aggregate data of non-collaborating studies and the pooled odds ratios of the IPD of collaborating studies was constructed for the primary outcome (live birth, intention to treat analysis).

Quality of the data and Integrity check

Data will be checked for missing values and entries that are outside the expected ranges, and internal consistency checks will be performed. The pattern of missing data will be analysed separately for each dataset in SPSS using the patterns chart. All variables will be selected besides the ID variable. We will analyze variables that are missing >0.01% data. First we will check which variables have missing data. Then, using the missing value pattern chart we will look for monotonicity. If missing data is concentrated in the upper left and lower right corner, there is a systematic pattern in missing data. If there are only patches or islands of missing values, the data is missing at a random pattern.

The quality of the data will be assessed by comparison of the provided IPD with the published baseline data and results. The baseline table will be reconstructed for each study, and baseline data checked for imbalances and overbalancing. The outcome data will be analyzed and the results compared with the published analyses. In case of any discrepancies or queries which may arise, the principal investigator will be contacted and corrections will be made if necessary.

Key design features and demographic characteristics of the included trials will be summarized in a table (Table 1).

Overall approach to IPD meta-analysis

We will use both ‘two-stage’ and ‘one-stage’ meta-analysis methods to synthesize the IPD.

We will conduct an initial two-stage IPD meta-analysis in order to obtain forest plots of the effect estimates and a measure of between-study heterogeneity. These intermediate results will inform the assumptions and choices for the final regression model.

Our primary analysis will be a one-stage IPD meta-analysis that will allow us to study both the treatment effect of endometrial scratching, and the hypothesized interactions with pre-specified patient-level characteristics in a single regression model.

**Type of analysis**

Primarily, an intention to treat analysis will be performed. Two types of secondary as treated (AT) analyses will be carried out as part of a sensitivity analysis strategy.

- Intention to treat (ITT):
  - All participants (i.e. also natural conceptions) will be included in the analysis and will be evaluated according to the treatment to which they were randomized.
  - The primary analysis consists of two counterparts:
    - Analysis of all included studies
    - Analysis according to the overall risk of bias:
      - Low risk only
      - low risk and some concerns
- As treated (AT):
  - All participants (i.e. also natural conceptions) will be analyzed according to the treatment they actually received.
- As treated and with embryo transfer (AT-ET):
  - Only participants with an embryo transfer in the treatment after randomization will be analyzed according to the treatment they actually received.

If participants became lost to follow-up or withdrew their informed consent before the intervention could have taken place, they will be excluded from all analyses.

Protocol violations, withdrawals, missing data, outliers and unexpected irregularities

Criteria

- Protocol violations:
  - Wrongfully included patients will be excluded from the full analysis set
  - Cross-over: patients receiving the treatment of the arm to which they were not randomized will primarily be analyzed according to the treatment to which they were allocated (i.e. intention to treat analysis). In the secondary as treated analysis, these patients will be analyzed in the arm corresponding to the treatment they actually received.
- Withdrawals:
  - Withdrawals will be included in the data analysis up to when the participant withdrew informed consent.
  - Missing covariate data after withdrawal of informed consent will be marked as ‘missing’ and imputed.
  - Participants with missing outcome data after withdrawal of informed consent will be excluded from analysis for the missing outcome(s).
- Lost to follow-up:
  - Data will be included until the time at which the participant became ‘lost to follow-up’ during the follow-up period of the study.
  - Missing covariate data for participants who became lost to follow-up will be marked as ‘missing’ and imputed.
  - Participants with missing outcome data after loss to follow-up will be excluded from analysis for the missing outcome(s).
- Missing data:
  - No available data at all for a participant: exclusion from the full analysis set.
  - Missing outcome data: exclusion from analysis for the missing outcome(s).
  - Sporadically missing covariate data: multiple imputation.
  - Entire variables missing from one study (systematically missing data): multiple imputation across studies. *NB: if an outcome variable was systematically not recorded by a study, that study was excluded from the analysis of that outcome.*
  - For further details on imputation see Methods for imputation of missing databelow
- Unexpected irregularities:
  - The strategy will be decided on when an unexpected irregularity is found in the dataset. This will be clearly described in the final manuscript.
- Outliers:
  - If outliers cannot be resolved, they will be marked as ‘missing’ and imputed.

The abovementioned is summarized in the following table and figure:

| **Category** | **Problem** | **Action** |
| --- | --- | --- |
| ***Protocol violation*** | Wrongfully included patients | Exclude from all analyses. |
|  | Cross-over | ITT analysis: analyze according to allocation  AT analysis: analyze according to received treatment |
| ***Withdrawals***  ***and***  ***loss to follow-up*** | Before the time point for which the scratch (or sham) was scheduled | Exclude from all analyses |
|  | After the time point for which the scratch (or sham) was scheduled, but before the first embryo transfer directly post-randomization | Include in ITT and AT analyses of outcomes of the first embryo transfer directly post-randomization, with all pregnancy outcomes = 0 |
|  | Any time point after the embryo transfer directly post-randomization | Include in all analyses of outcomes for which outcome data is available |
| ***Missing data*** | No available data at all | Exclude |
|  | Sporadically/randomly missing data | Determine if MCAR, MAR, MNAR  Multiple imputation within studies |
|  | Systematically missing data | Determine if MCAR, MAR, MNAR  Multiple imputation across studies |
| ***Unexpected irregularities*** | Not yet known | To be determined on a case-by-case basis |
| ***Outliers*** | Outliers will primarily be tried to resolve. It may sometimes be impossible to resolve. | Mark as missing and impute |

**Criteria for handling protocol violations, missing data, outliers and unexpected irregularities in the datasets.** Abbreviations: MCAR = missing completely at random; MAR = missing at random; MNAR = missing not at random

**Criteria for handling withdrawals and loss to follow-up at different time points in the studies**

Methods for imputation of missing data

- We will use the ‘MICE’ R package to perform multiple imputation of missing covariate data.
- Sporadically missing data (i.e. a study recorded this data for all its patients, but it was missing for some patients):
  - Will be imputed *within each study* by means of an imputation mixed model (multilevel imputation), using study ID as clustering variable.
  - Each variable that contains sporadically missing data will be evaluated separately whether imputation can be performed or whether the level of missingness is too high. As the quality of imputation depends on the characteristics of the variable, it is not possible to apply a single cut-off percentage of missingness for all variables.
- Systematically missing data (i.e. a study systematically did not record this data):
  - Will be imputed *across studies* by means of an imputation mixed model (multilevel imputation), using study ID as clustering variable.
  - Each variable with systematically missing data will be evaluated separately if imputation can be performed or if the level of missingness is too high. As the quality of imputation depends on the characteristics of the variable, it is not possible to apply a single cut-off percentage of missingness for all variables.
- Multiple imputation techniques assume MAR (missing at random) pattern. If data is MNAR (missing not at random) or MCAR (missing completely at random), we will conduct a sensitivity analysis in order to assess the robustness of the imputed datasets under different hypotheses for the missingness pattern.

Baseline characteristics

Methods

- Baseline tables will be constructed separately for each studied outcome and will display only the baseline characteristics of those participants included in the analysis.
- In all cases where we aim to report a mean (±SD), a median with interquartile ranges will be given instead of mean with standard deviation if the variable is non-normally distributed. Binary and categorical variables will be presented as the number of total and percentage.
- No statistical test will be performed to determine if the groups are significantly different from each other or not, as we assume any baseline differences after randomization have occurred due to chance.
- Overall baseline characteristics will be presented per group (control versus intervention, see Table 2).

Missing values

- The number of missing values that were imputed will be indicated in the table.

Variables

- The following variables will be reported^[1]^:
- Female age in years, mean ±SD
- Female Body Mass Index in kg/m^2^, mean ±SD
- Female smoking, n (%)
- Female gravidity, n (%)
  - 0
  - 1
  - ≥2
- Female parity, n (%)
  - 0
  - 1
  - ≥2
- Type of infertility of the female
  - Primary, n (%)
  - Secondary, n (%)
- Type of infertility of the couple *Post-hoc: this data was unavailable in almost all studies*
  - Primary, n (%)
  - Secondary, n (%)
- Duration of infertility of the couple in months, mean ±SD
- Cause of infertility *Post-hoc: in order for imputation to be possible, this variable had to be reduced to three categories: male factor, unexplained and other.*
  - Idiopathic, n (%)
  - Male factor, n (%)
  - Tubal factor, n (%)
  - Ovulatory disorder, n (%)
  - Endometriosis grade 1-2, n (%)
  - Endometriosis grade 3-4, n (%)
  - Other (including lesbian couple, single women), n (%)
- Number of previous failed^[2]^ embryo transfers

Treatment outcomes

We will study the effect of treatment for the following outcomes:

Primary

Live birth (i.e. birth of a live foetus from 24 weeks of gestation onwards) from the first treatment following randomisation per woman randomized. If live birth is not reported, ongoing pregnancy (defined as a positive foetal heartbeat on ultrasound from a gestational age of 10 weeks onwards) will be used as a proxy.

*Note:* Multiple live births (e.g. twins or triplets) will be counted as one live birth event. Similarly, the presence of multiple foetal heartbeats on ultrasound from a gestational age of 10 weeks onwards will be counted as one ongoing pregnancy.

Secondary

- Cumulative live birth from pregnancies that achieved a positive pregnancy test within 6 months following randomisation Post-hoc: only 2 trials systematically recorded data up until 6 months post-randomisation or beyond. We therefore maintained the 6 month time-limit for treatments and pregnancies, but did not report cumulative live birth outcomes.
- Ongoing pregnancy from the embryo transfer directly after randomisation.
- Clinical pregnancy from the embryo transfer directly after randomization. Clinical pregnancy is defined as the presence of a gestational sac on ultrasound at a gestational age of 6-7 weeks. Multiple gestational sacs on ultrasound at a gestational age of 6-7 weeks will be counted as one clinical pregnancy.
- Miscarriage from the treatment directly after randomization. In the case of a multiple pregnancy, a miscarriage is defined as the loss of all fetuses.
- Ectopic pregnancy from the treatment directly after randomization.
- Multiple pregnancy from the treatment directly after randomization. Multiple pregnancy is defined as the live birth or ongoing pregnancy of two or more fetuses.

Additionally, for the intervention group, the outcomes ‘Pain during the procedure (visual analogue scale (VAS) or Numeric rating scale (NRS) score)’ and ‘Adverse events (e.g. infection)’ will be analysed using descriptive statistics (numbers and % of total).

These outcomes and their corresponding variables and possible values are summarized in the following table:

| **Outcome** | **Type** | **Values** | **Description** |
| --- | --- | --- | --- |
| Comparisons | | | |
| *Primary* | | | |
| C1_LBorOP | Binary | 0 = "No"  1 = "Yes" | Combined outcome  = C1_LB if available  (live birth of embryo transfer directly post-randomisation)  = C1_OP otherwise  (ongoing pregnancy of embryo transfer directly post-randomisation) |
| *Secondary* | | | |
| C2_LBorOP | Binary | 0 = "No"  1 = "Yes" | Combined outcome  = C2_LB if available  = C2_OP otherwise  Cumulative live birth or ongoing pregnancy after embryo transfer that took place within 6 months post-randomisation |
| C1_OP | Binary | 0 = "No"  1 = "Yes" | Ongoing pregnancy of embryo transfer directly post-randomisation |
| C1_CP | Binary | 0 = "No"  1 = "Yes" | Clinical pregnancy of embryo transfer directly post-randomisation |
| C1_Misc | Binary | 0 = "No"  1 = "Yes" | Miscarriage of embryo transfer directly post-randomisation |
| C1_EUG | Binary | 0 = "No"  1 = "Yes" | Ectopic pregnancy of embryo transfer directly post-randomisation |
| C1_OPmultBin | Binary | 0 = "No"  1 = "Yes | Multiple live birth or ongoing pregnancy of embryo transfer directly post-randomisation |
| Descriptive analysis intervention group | | | |
| Scr_Pain | Continuous | 0 - 10 | Pain score during scratch (VAS or NRS from 0-10, 0=no pain, 10=most pain. If available: prove score with 1 decimal) |
| Scr_AE | Categorical | 0 = “No”  1 = “Bleeding”  2 = “Abdominal pain”  3 = “Infection”  4 = “Uterine perforation”  5 = “Other” | Were any symptoms reported by the patient in the week after the scratch? |

**Summary of the outcome variables**

Effect modifiers (subgroup analyses)

Effect modifiers will be studied on the AT-ET population only. We will study the following hypothesized patient-level modifiers of treatment outcome:

- Number of previously unsuccessful embryo transfers:
  - Unsuccessful is defined as not achieving a live birth.
  - The number of previously unsuccessful embryo transfers will be recorded as such for each patient, and will be analysed as a continuous variable.
- Type of treatment:
  - Recorded in detail for each patient, but summarized for analysis in the dichotomized variable TyTr (fresh vs. cryo transfer).
- Timing of endometrial scratch:
  - Follicular phase prior cycle vs. mid-cyclic prior cycle vs. luteal phase prior cycle vs. follicular phase stimulation cycle
  - Post-hoc analysis: the originally planned analysis appeared to be impossible due to too little within-study variation, too little individual patient data on the menstrual-cycle-timing and the inability to impute this reliably. We therefore decided to perform a conventional subgroup analysis comparing studies that applied luteal phase scratching to studies with other timing.

These interactions and their corresponding variables and possible values are summarized in the following table:

| **Variable** | **Type** | **Values** | **Description** |
| --- | --- | --- | --- |
| No_ETs | Continuous | Scale | The total number of previous unsuccessful embryo transfers. |
| TyTr | Binary | 0 = “Fresh”  1 = “Cryo” | Fresh (IVF or ICSI) vs. cryo transfer |
| Scr_tim | Categorical | 0 = "Follicular of pre-transfer cycle"  1 = “Mid-cyclic of pre-transfer cycle”  2 = "Luteal of pre-transfer cycle "  3 = "Follicular of transfer cycle" | Timing of the scratch |

**Hypothesized interactions (effect modifiers, subgroups) that will be studied**

Post-hoc analysis:

During the data-cleaning process but before starting analysis, we decided to add analysis of a few other possible effect modifiers based on previously published hypotheses. The additional patient-level modifiers of treatment-effect outcome are:

- Age at randomisation (continuous)
- Infertility cause:
  - Unexplained vs. Male factor vs. Other
    - ‘Other’ includes ovulatory disorders, tubal factor, endometriosis, mixed diagnosis

STATISTICAL ANALYSIS II: PRELIMINARY TWO-STAGE META-ANALYSIS

Step 1

For each included study, the outcomes listed as ‘Comparisons’ in the Outcome**s** table above will be compared between the treatment and control groups in a 2x2 table, and odds ratios with 95% confidence intervals will be calculated.

Step 2

The generated summary estimates will be combined using a random-effects model, as we assume differences in treatment effect due to between-study heterogeneity.

- The Paule-Mandel method will be used to estimate the heterogeneity variance τ^2^ (Langan, Higgins and Simmonds, 2017).
- Confidence intervals will be calculated using a Student’s t-distribution and adjusting the standard error of the summary estimate using the Hartung-Knapp-Sidik-Jonkman correction (IntHout, Ioannidis and Borm, 2014).
- We will calculate 95% prediction intervals.
- The results will be presented in forest plots.
- The I^2^ statistic will be calculated to provide an additional measure of between-study heterogeneity.

STATISTICAL ANALYSIS III: PRIMARY ONE-STAGE META-ANALYSIS

Fit generalized linear mixed regression model (GLMM) to the outcomes listed as ‘Comparisons’ in the Outcome**s** table above, with:

- GLMM: logistic regression (outcomes are binary) to obtain odds ratios
- Random effects for the intercepts.
- Random effects for treatment effect.
- Random effects for treatment-covariate interaction terms and confounders.
  - Separate within- and between-trial interaction terms, centering covariates around the average within each study (as opposed to the overall average).
  - Specify beforehand all covariates to adjust for, i.e.:
    - Known confounders, e.g.:
      - Baseline imbalances despite randomization;
      - Different inclusion and exclusion criteria (e.g. age);
      - Study design (e.g. length of follow-up);
    - Interactions (effect modifiers) that we want to explore:
      - Type of treatment (binary);
      - Number of failed embryo transfers (continuous);
      - Timing of the scratch (categorical);
      - Age (continuous);
      - Infertility cause (categorical).

Bonferroni correction for multiple hypotheses.

STATISTICAL ANALYSIS IV: DESCRIPTIVE ANALYSIS INTERVENTION GROUP

Outcome: Pain

*Methods:*

- Mean and standard deviation (SD) will be calculated in case of a normal distribution
- In case of non-normal distribution, median and interquartile range (IQR) will be calculated
- Analysis will be performed on an all women that truly received endometrial scratching. This includes the population in which the procedure was started but not completed (failure to complete the intervention).

Outcome: Adverse events

*Methods:*

- The number of patients with an adverse event (AE) in the week following endometrial injury will be reported compared to the total number of participants that received endometrial scratching (the intervention). This includes the population in which the procedure was started but not completed (failure to complete the intervention).

EXAMPLE TABLES & FIGURES

Table 1. Design features and demographic characteristics of included RCTs

|  | **Country** | **Recruit-ment period** | **Type of treatment** | **Study population** | **Device used for scratching** | **Furthest stage of pregnancy reported as outcome** | **Number of patients** | **Female mean age (range; years)** | **Female mean BMI (range; kg/m^2^)** | **Mean duration of infertility (range; months)** | **Number of failed embryo transfers** |
| --- | --- | --- | --- | --- | --- | --- | --- | --- | --- | --- | --- |
| **Aflatoonian et al** | Iran | 2015-16 | FET | undergoing FET | pipelle | OPR | 93 | mean (range) | mean (range) | mean (range) | n (%) |
| **Aleyamma et al** | India | 2008-15 | IVF | at least one previous failed cycle with minimum of two good quality embryos transferred in an earlier attempt | pipelle | LBR | 111 | mean (range) | mean (range) | mean (range) | n (%) |
| **Baum et al** | Israel | 2006-09 | IVF | three or more unsuccessful cycles of IVF-Embryo Transfer (ET) with good ovarian response in previous cycles | pipelle | LBR | 36 | mean (range) | mean (range) | mean (range) | n (%) |
| **…** | … | … | … | … | … | … | … | … | … | … | … |

Table 1. Design features and demographic characteristics of included RCTs

Table 2. Overall baseline characteristics

| **Total participants in analysis** | **Scratch (n)** | **Control (n)** |
| --- | --- | --- |
| **Female age (years)** | mean (±SD) [# imputed] | mean (±SD) [# imputed] |
| **Female BMI (kg/m^2^)** | mean (±SD) [# imputed] | mean (±SD) [# imputed] |
| **Duration of infertility (months)** | mean (±SD) [# imputed] | mean (±SD) [# imputed] |
| **…** |  |  |

Table 2. Overall baseline characteristics of included participants

Table 3. Descriptive analysis

| **Total participants in analysis** | | **Scratch (n)** | **Control (n)** |
| --- | --- | --- | --- |
| Number of patients that truly received treatment to which they were allocated | | n | n |
| **1^st^ IVF/ICSI/FET treatment post-randomization** | | | |
|  | Fresh IVF/ICSI | n (%) | n (%) |
|  | FET | n (%) | n (%) |
| Number of embryos transferred | |  |  |
|  | No transfer | n (%) | n (%) |
|  | SET | n (%) | n (%) |
|  | DET | n (%) | n (%) |
|  | TET | n (%) | n (%) |
|  | QET or more | n (%) | n (%) |
| **Participants followed-up after failed 1^st^ IVF/ICSI/FET treatment post-randomization** | | | |
|  | Number of participants | n (%) | n (%) |
|  | Mean duration of follow-up | mean (±SD) | mean (±SD) |
|  | Total number of embryo  transfers during follow-up | mean (±SD) | mean (±SD) |

Tabel 1 Descriptive analysis of treatment and follow-up Abbreviations SET: single embryo transfer, DET: double embryo transfer, TET: triple embryo transfer, QET: quadruple embryo transfer

Table 4. Treatment effects on primary and secondary pregnancy outcomes: results of one-stage model (1)

|  | Scratch (n) | Control (n ) | OR | (95%CI) | (PI) |
| --- | --- | --- | --- | --- | --- |
| **Primary outcome** |  |  |  |  |  |
| Live birth from the first treatment after randomization | n (%) | n (%) |  |  |  |
| **Secondary outcomes** |  |  |  |  |  |
| Cumulative live birth after 6 months following randomisation | n (%) | n (%) |  |  |  |
| Ongoing pregnancy from the treatment directly after randomisation | n (%) | n (%) |  |  |  |
| Clinical pregnancy from the treatment directly after randomization | n (%) | n (%) |  |  |  |
| Miscarriage from the treatment directly after randomization | n (%) | n (%) |  |  |  |
| Ectopic pregnancy from the treatment directly after randomization | n (%) | n (%) |  |  |  |
| Multiple pregnancy from the treatment directly after randomization | n (%) | n (%) |  |  |  |

Tabel 2 Estimated treatment effects of endometrial scratching for the studied primary and secondary pregnancy outcomes

Table 5. Effect modifiers (subgroup analyses): results of one-stage model (2)

| **Hypothesized effect modifier** | | **Interaction effect** | **p value** |
| --- | --- | --- | --- |
| **Number of failed embryo transfers** | |  |  |
| **Type of treatment (fresh IVF/ ICSI vs. cryo transfer)** | |  |  |
| **Timing of the scratch** | |  |  |
|  | **Follicular of pre-transfer cycle** |  |  |
|  | **Luteal of pre-transfer cycle** |  |  |
|  | **Follicular of transfer cycle** |  |  |

Tabel 3 Estimated interaction effects for the hypothesized effect modifiers

Table 6. Pain and adverse events in the week after scratch (scratch group only)

| **Adverse event** | Scratch (n) |
| --- | --- |
| Bleeding | n (%) |
| Watchful waiting |  |
| Needed outpatient treatment |  |
| Needed hospitalization |  |
| Other |  |
| Abdominal pain | n (%) |
| Watchful waiting |  |
| Needed outpatient treatment |  |
| Needed hospitalization |  |
| Other |  |
| Infection | n (%) |
| Watchful waiting |  |
| Needed outpatient treatment |  |
| Needed hospitalization |  |
| Other |  |
| Uterine perforation | n (%) |
| Watchful waiting |  |
| Needed outpatient treatment |  |
| Needed hospitalization |  |
| Other |  |
| Other | n (%) |
| Watchful waiting |  |
| Needed outpatient treatment |  |
| Needed hospitalization |  |
| Other |  |
| **Pain** |  |
| VAS or NRS | Mean (±SD) |
|  |  |

Figure 1. PRISMA-IPD flow diagram

Figure 1 PRISMA-IPD flow diagram

Figure 2. Risk of bias of eligible studies

**Studies that provided IPD**

**Studies that did not provide IPD**

Figure 2 Risk of bias of eligible studies

Figure 3. Funnel plot for the primary outcome (LBR)

 Figure 3 Funnel plot of eligible studies

Baum 2012

Singh 2015

Lensen 2019

Karimzade 2010

Mahran 2016

Figure 4. Forest plot for primary outcome: live birth rate

Figure 5. Forest plot for cumulative LBR

**Figure 6. Forest plot for** **OPR**

Figure 7. Forest plot for CPR

Figure 8. Forest plot for miscarriage

Figure 9. forest plot for multiple pregnancy

Figure 10. Forest plot for ectopic pregnancy

REFERENCES

Cao, H. *et al.* (2018) ‘Hysteroscopy after repeated implantation failure of assisted reproductive technology: A meta-analysis’, *Journal of Obstetrics and Gynaecology Research*, 44(3), pp. 365–373. doi: 10.1111/jog.13571.

De Geyter, C. *et al.* (2018) ‘ART in Europe, 2014: results generated from European registries by ESHRE†’, *Human Reproduction*, 33(9), pp. 1586–1601. doi: 10.1093/humrep/dey242.

Gui, J. *et al.* (2019) ‘Impact of local endometrial injury on in vitro fertilization/intracytoplasmic sperm injection outcomes: A systematic review and meta-analysis’, *Journal of Obstetrics and Gynaecology Research*, 45(1), pp. 57–68. doi: 10.1111/jog.13854.

van Hoogenhuijze, N. E. *et al.* (2019) ‘Endometrial scratching prior to IVF; does it help and for whom? A systematic review and meta-analysis’, *Human Reproduction Open*, 2019(1), pp. 1–18. doi: 10.1093/hropen/hoy025.

IntHout, J., Ioannidis, J. P. A. and Borm, G. F. (2014) ‘The Hartung-Knapp-Sidik-Jonkman method for random effects meta-analysis is straightforward and considerably outperforms the standard DerSimonian-Laird method.’, *BMC medical research methodology*. BMC Medical Research Methodology, 14, p. 25. doi: 10.1186/1471-2288-14-25.

Langan, D., Higgins, J. P. T. and Simmonds, M. (2017) ‘Comparative performance of heterogeneity variance estimators in meta-analysis: a review of simulation studies’, *Research Synthesis Methods*, 8(2), pp. 181–198. doi: 10.1002/jrsm.1198.

Nastri, C. O. *et al.* (2015) ‘Endometrial injury in women undergoing assisted reproductive techniques.’, *The Cochrane database of systematic reviews*, (3), p. CD009517. doi: 10.1002/14651858.CD009517.pub3.

Panagiotopoulou, N., Karavolos, S. and Choudhary, M. (2015) ‘Endometrial injury prior to assisted reproductive techniques for recurrent implantation failure: a systematic literature review’, *European Journal of Obstetrics & Gynecology and Reproductive Biology*. Elsevier Ireland Ltd, 193, pp. 27–33. doi: 10.1016/j.ejogrb.2015.06.026.

Riley, R. D., Lambert, P. C. and Abo-Zaid, G. (2010) ‘Meta-analysis of individual participant data: rationale, conduct, and reporting.’, *BMJ (Clinical research ed.)*, 340(7745), p. c221. doi: 10.1136/bmj.c221.

Sterne, J. A. C. *et al.* (2019) ‘RoB 2: A revised tool for assessing risk of bias in randomised trials’, *The BMJ*, 366, pp. 1–8. doi: 10.1136/bmj.l4898.

Stewart, L. A. *et al.* (2015) ‘Preferred Reporting Items for Systematic Review and Meta-Analyses of individual participant data: the PRISMA-IPD Statement.’, *JAMA*, 313(16), pp. 1657–65. doi: 10.1001/jama.2015.3656.

Tierney, J. F. *et al.* (2015) ‘Individual Participant Data (IPD) Meta-analyses of Randomised Controlled Trials: Guidance on Their Use’, *PLOS Medicine*, 12(7), p. e1001855. doi: 10.1371/journal.pmed.1001855.

Vitagliano, A. *et al.* (2018) ‘Endometrial scratch injury before intrauterine insemination: is it time to re-evaluate its value? Evidence from a systematic review and meta-analysis of randomized controlled trials’, *Fertility and Sterility*, 109(1), pp. 84-96.e4. doi: 10.1016/j.fertnstert.2017.09.021.

Vitagliano, A. *et al.* (2019) ‘Endometrial scratching for infertile women undergoing a first embryo transfer: a systematic review and meta-analysis of published and unpublished data from randomized controlled trials’, *Fertility and Sterility*. Elsevier Inc. doi: 10.1016/j.fertnstert.2018.12.008.

1. **We aim to report these variables, but are dependent on what is reported in the original datasets. Therefore, we may have to adjust certain variables to represent the received data better. This can be expected for variables such as ‘cause of infertility’ because of the lack of use of a single, uniform definition across countries.*
2. Failed meaning: not having reached live birth
