# Supplementary material for: Endometrial scratching in women undergoing IVF/ICSI: an individual participant data meta-analysis
Source: Hum Reprod Update. 2023 Jun 19;29(6):721–40. doi: 10.1093/humupd/dmad014 (PMC10628489; doi:10.1093/humupd/dmad014)
Supplement: dmad014_Supplementary_Data [file dmad014_supplementary_data.zip › dmad014_Supplementary_Data/Supplementary Tables and Figures EO.doc]

| **Supplementary Table S1.** Search strategy. | |
| --- | --- |
| **Database** | **Search string** |
| Pubmed | (("ART"[Title/Abstract] OR "assisted conception"[Title/Abstract] OR "assisted reproduction"[Title/Abstract] OR "IVF"[Title/Abstract] OR "in vitro fertilisation"[Title/Abstract] OR "in vitro fertilization"[Title/Abstract] OR "intracytoplasmic sperm injection"[Title/Abstract] OR "ICSI"[Title/Abstract] OR "*Embryo Transfer"[Title/Abstract] OR "ET"[Title/Abstract] OR "frozen embryo transfer"[Title/Abstract] OR "FET"[Title/Abstract] OR "implantation failure"[Title/Abstract] OR "recurrent implantation failure"[Title/Abstract] OR "subfertility"[Title/Abstract])) AND ("endometrial biopsy"[Title/Abstract] OR "endometrial injury"[Title/Abstract] OR "endometrial trauma"[Title/Abstract] OR "mock embryo transfer"[Title/Abstract]) |
|  |  |
| Embase | “ART” or “assisted conception” or “assisted reproduction” or “IVF” or “in vitro fertilisation” or “in vitro fertilization” or “intracytoplasmic sperm injection” or “ICSI” or “ *Embryo Transfer” or “ET” or “frozen embryo transfer” or “FET” or “implantation failure” or “recurrent implantation failure” or “subfertility” AND “endometrial biopsy” or “endometrial injury” or “endometrial trauma” or “mock embryo transfer” |
|  |  |
| Cochrane | “ART” or “assisted conception” or “assisted reproduction” or “IVF” or “in vitro fertilisation” or “in vitro fertilization” or “intracytoplasmic sperm injection” or “ICSI” or “ *Embryo Transfer” or “ET” or “frozen embryo transfer” or “FET” or “implantation failure” or “recurrent implantation failure” or “subfertility” AND “endometrial biopsy” or “endometrial injury” or “endometrial trauma” or “mock embryo transfer” |
|  |  |
| Clinicaltrials.gov | 'endometrial scratching' OR 'endometrial injury' OR 'endometrial pipelle' OR 'endometrial biopsy' OR 'endometrial trauma' OR 'endometrial disruption' OR 'endometrial inflammation' |
| Syntax used for systematic search in various electronic databases. | |

|  | **Supplementary Table S2.** Overview of non-participating studies. | | | |
| --- | --- | --- | --- | --- |
| **Study** | | **Country** | **Registration number** | **Reason not to participate** |
|  | ***Published studies (full text or conference abstract) – no or negative response*** | | | |
| Aflatoonian 2016 | | Iran | N.A. | Agreed to participate, but did not receive responses thereafter. |
| Baum 2012 | | Israel | N.A. | Never received a response. |
| Eskew 2018 | | USA | N.A. | Agreed to participate, but did not receive responses thereafter. |
| Frantz 2018 | | France | NCT01064193 | Author’s response: ‘No’. |
| Gibreel 2015 | | Egypt | NCT01245309 | Agreed to participate, but after discussion with co-authors decided not to participate. |
| Guven 2014 | | Turkey | NCT01851876 | Agreed to participate, but did not receive responses thereafter. |
| Hilton 2016 | | Canada | NCT01983423 | Agreed to participate, but did not receive responses thereafter. |
| Hur 2012 | | South Korea | N.A. | Author’s response: ‘Have lost the raw data’. |
| Karimzade 2010 | | Iran | NCT00846183 | Never received a response |
| Karimzadeh 2009 | | Iran | NCT00837733 ^a^ | Never received a response. |
| Karim Zadeh Meybodi 2008 | | Iran | N.A. | Never received a response. |
| Liu 2017 | | China | ChiCTR-IOR-17011506 | IPD should be publically available on medresman.org but could not be found. Did not receive a response from the authors. |
| Maged 2017 | | Egypt | NCT02660125 | Author’s response: ‘No time’. |
| Mehrafza 2010/2011 | | Iran | N.A. | Never received a response. |
| Pecorino 2018 | | Italy | N.A. | Agreed to participate, but did not receive responses thereafter. |
| Safdarian 2011 | | Iran | IRCT201008154572N1 | Never received a response. |
| Shahrokh-Tehraninejad 2016 | | Iran | IRCT201311065181N12R2 | Never received a response. |
| Sherif 2018 | | Egypt | PACTR201701001968212 | Never received a response. |
| Shohayeb 2014 | | Egypt, SA | N.A. | Never received a response. |
| Singh 2015 | | India | N.A. | Agreed to participate, but did not receive responses thereafter. |
| Zhao 2015 | | China | N.A. | Never received a response. |
|  | ***Published studies – inability to share*** | | |  |
| Metwally 2020 | | UK | ISRCTN23800982 | Author agreed to participate in the future. Cannot share data before all dissemination activities have been completed. |
|  | ***Registered not published studies*** *^b^* | | | |
| Abdelaziz 2016 | | Egypt | NCT02752568 | Stated that they are not planning to share data. |
| Aghajanpour 2015 | | Iran | NCT02480127 | Never received a response. Also: unclear if the required pregnancy outcomes were recorded. |
| Baum 2006 | | Israel | NCT00411021 | Never received a response. |
| Barash 2011 | | Israel | NCT01278706 | Author’s response: ‘Don’t have data’. |
| Elbohoty 2014 | | Egypt | NCT02084914 | Never received a response. |
| Genod 2015 | | France | NCT02522806 | Never received a response. |
| Li 2014 | | China | NCT02306395 | Never received a response. |
| Martins 2014 | | Brazil | NCT02180256 | Author’s response: Stopped recruitment very early, did not record any data. |
| Martins 2014 | | Brazil | NCT02093442 | Author’s response: Stopped recruitment very early, did not record any data. |
| Mohammed 2014 | | Egypt | NCT02245750 | Never received a response. |
| Nayot 2013 | | Canada | NCT01844453 | Author’s response: Never started recruitment. |
| Rose 2017 | | USA | NCT03157765 | Author’s response: Never started recruitment. |
| Sherbiny 2017 | | Egypt | NCT03220503 | Never received a response. |
| Sun 2015 | | China | NCT02409745 | Never received a response. |
| Wolff 2013 | | USA | NCT01800513 | Agreed to participate, but did not receive responses thereafter. |
| N.A., not available; SA, Saudi Arabia; UK, United Kingdom; USA, United States of America.  ^a^ We assume that this NCT number corresponds to the study of Karimzadeh 2009 but are not completely sure.  ^b^ The year indicates the year of first registration | | | | |

| **Supplementary Table S3.** Baseline characteristics of included participants – AT analysis. | | | | | |
| --- | --- | --- | --- | --- | --- |
| **Total participants in analysis** | | **Received Scratch**  **(n= 1,909)** | | **Received Control**  **(n= 2,043)** | |
| **Randomised for** | |  |  |  |  |
|  | Scratch | 1,904 | (99.7%) | 1,968 | (96.3%) |
|  | Control | 5 | (0.3%) | 75 | (3.5%) |
| **Female age (years) ^a^** | | 34.7 | (31.0 – 38.0) | 35.0 | (31.5 – 38.0) |
| **Female BMI (kg/m^2^) ^b^** | | 23.3 | (21.0 – 26.4) | 23.6 | (21.3 – 26.8) |
| **Duration of infertility (months) ^c^** | | 36.0 | (24.0 – 59.0) | 36.0 | (24.0 – 60.0) |
| **Number of previous failed embryo transfers ^d^** | | | |  |  |
|  | 0 | 522 | (27.3%) | 587 | . |
|  | 1 | 506 | (26.5%) | 623 | (30.5%) |
|  | 2 | 364 | (19.1%) | 321 | (15.7%) |
|  | 3 | 174 | (9.1%) | 182 | (8.9%) |
|  | ≥4 | 205 | (10.7%) | 203 | (9.9%) |
| **Smoking status of the female ^e^** | |  |  |  |  |
|  | No | 1,383 | (72.4%) | 1,508 | (73.8%) |
|  | Yes | 101 | (5.3%) | 107 | (5.2%) |
|  | Quit | 10 | (0.5%) | 6 | (0.3%) |
| **Gravidity of the female ^f^** | |  |  |  |  |
|  | 0 | 772 | (40.4%) | 820 | (40.1%) |
|  | 1 | 367 | (19.2%) | 403 | (19.7%) |
|  | 2 | 139 | (7.3%) | 154 | (7.5%) |
|  | ≥3 | 95 | (5.0%) | 119 | (5.8%) |
| **Parity of the female ^g^** | |  |  |  |  |
|  | 0 | 707 | (37.0%) | 771 | (37.7%) |
|  | 1 | 152 | (8.0%) | 135 | (6.6%) |
|  | 2 | 19 | (1.0%) | 14 | (0.7%) |
|  | ≥3 | 6 | (0.3%) | 8 | (0.4%) |
| **Primary infertility of the female ^h^** | |  |  |  |  |
|  | No | 634 | (33.2%) | 708 | (34.7%) |
|  | Yes | 1,020 | (53.4%) | 1,080 | (52.9%) |
| **Cause of infertility ^i^** | |  |  |  |  |
|  | Unexplained | 456 | (23.9%) | 474 | (23.2%) |
|  | Male factor | 455 | (23.2%) | 483 | (23.6%) |
|  | Tubal | 356 | (18.6%) | 384 | (18.8%) |
|  | Endometriosis | 108 | (5.7%) | 128 | (6.3%) |
|  | Ovulatory disorder | 168 | (8.8%) | 190 | (9.3%) |
|  | Other / Mixed diagnosis | 123 | (6.4%) | 134 | (6.6%) |
| Data are median(IQR) or n (% of randomized population). AT: as treated; BMI: body mass index. 12 studies (3,954 participants). Observed data only. Adherence to allocated treatment (i.e. scratch performed or not) was missing for 2 participants. These participants are *not* represented in this table, but are included in the analyses for treatment effect (Table S5) with imputed data for adherence to allocation.  ^a^ Data is missing for 9 and 12 participants, resp.  ^b^ Data is missing for 44 and 55 participants, resp.  ^c^ Data is missing for 473 and 476 participants, resp.  ^d^ Data is missing for 138 and 127 participants, resp.  ^e^ Data is missing for 415 and 422 participants, resp.  ^f^ Data is missing for 536 and 547 participants, resp.  ^g^ Data is missing for 1,115 and 1,025 participants, resp.  ^h^ Data is missing for 225 and 225 participants, resp.  ^i^ Data is missing for 243 and 250 participants, resp. | | | | | |

| **Supplementary Table S4.** Baseline characteristics of included participants – AT+ET analysis. | | | | | |
| --- | --- | --- | --- | --- | --- |
| **Total participants in analysis** | | **Received Scratch and ET**  **(n= 1,612 )** | | **Received Control and ET**  **(n= 1,625)** | |
| **Randomised for** | |  |  |  |  |
|  | Scratch | 1,607 | (99.7%) | 1,583 | (97.4%) |
|  | Control | 5 | (0.3%) | 42 | (2.6%) |
| **Female age (years) ^a^** | | 35.0 | (31.3 – 38.0) | 35.0 | (31.8 – 38.0) |
| **Female BMI (kg/m^2^) ^b^** | | 23.2 | (21.0 – 26.2) | 23.4 | (21.2 – 26.5) |
| **Duration of infertility (months) ^c^** | | 36..0 | (24.0 – 57.0) | 36.0 | (24.0 – 60.0) |
| **Number of previous failed embryo transfers ^d^** | | | |  |  |
|  | 0 | 431 | (26.7%) | 447 | (27.5%) |
|  | 1 | 439 | (27.2%) | 490 | (30.2%) |
|  | 2 | 313 | (19.4%) | 269 | (16.6%) |
|  | 3 | 154 | (9.6%) | 157 | (9.7%) |
|  | ≥4 | 174 | (10.8%) | 167 | (10.3%) |
| **Smoking status of the female ^e^** | |  |  |  |  |
|  | No | 1,160 | (72.0%) | 1,173 | (72.2%) |
|  | Yes | 81 | (5.0%) | 85 | (5.2%) |
|  | Quit | 10 | (0.6%) | 5 | (0.3%) |
| **Gravidity of the female ^f^** | |  |  |  |  |
|  | 0 | 646 | (40.1%) | 620 | (38.2%) |
|  | 1 | 317 | (19.7%) | 312 | (19.2%) |
|  | 2 | 126 | (7.8%) | 119 | (7.4%) |
|  | ≥3 | 83 | (5.1%) | 105 | (6.5%) |
| **Parity of the female ^g^** | |  |  |  |  |
|  | 0 | 613 | (38.0%) | 641 | (39.4%) |
|  | 1 | 136 | (8.4%) | 110 | (6.8%) |
|  | 2 | 16 | (1.0%) | 9 | (0.6%) |
|  | ≥3 | 5 | (0.3%) | 6 | (0.4%) |
| **Primary infertility of the female ^h^** | |  |  |  |  |
|  | No | 547 | (33.9%) | 572 | (35.2%) |
|  | Yes | 855 | (53.0%) | 841 | (51.8%) |
| **Cause of infertility ^i^** | |  |  |  |  |
|  | Unexplained | 391 | (24.3%) | 361 | (22.2%) |
|  | Male factor | 395 | (24.5%) | 405 | (24.9%) |
|  | Tubal | 306 | (19.0%) | 295 | (18.2%) |
|  | Endometriosis | 93 | (5.8%) | 98 | (6.0%) |
|  | Ovulatory disorder | 125 | (7.8%) | 146 | (9.0%) |
|  | Other / Mixed diagnosis | 107 | (6.6%) | 113 | (7.0%) |
| Data are median(IQR) or n (% of randomized population). AT+ET: as treated with embryo transfer; BMI: body mass index.  Data is based on 12 studies (3,954 participants) of whom the participants who underwent embryo transfer were selected. Data on receiving an embryo transfer was imputed for 19 participants, therefore the number of included participants for the analyses varied between 3,250 and 3,257 (mean=3,254).  Shown numbers and medians are observed data only. The outcome analysis was performed on imputed data.  ^a^ Data is missing for 2 and 2 participants, resp.  ^b^ Data is missing for 30 and 33 participants, resp.  ^c^ Data is missing for 390 and 397 participants, resp.  ^d^ Data is missing for 101 and 95 participants, resp.  ^e^ Data is missing for 361 and 362 participants, resp.  ^f^ Data is missing for 447 and 462 participants, resp.  ^g^ Data is missing for 842 and 859 participants, resp.  ^h^ Data is missing for 210 and 212 participants, resp.  ^i^ Data is missing for 195 and 207 participants, resp. | | | | | |

**Supplementary Figure S1:** Forest plot of two-stage As Treated analysis, outcome: Live Birth.

AT: as treated; OR: odds ratio; 95% CI: 95% confidence interval. 12 studies (3,954 participants).

Shown event numbers are observed values only. The per-study and pooled odds ratios are based on imputed data. Whether a scratch was performed was imputed for 2 participants.

The outcome live birth was imputed for 100 participants (fraction of missing outcome data 2.5%).

**Supplementary Figure S2:** Forest plot of two-stage As Treated with Embryo Transfer analysis, outcome: Live Birth.

AT+ET; as treated with embryo transfer; OR, odds ratio; 95% CI: 95% confidence interval. 12 studies.

Shown event numbers are observed values only. The per-study and pooled odds ratios are based on imputed data.

Analysis was restricted to those patients receiving an embryo transfer. Data on embryo transfer was imputed for 19 participants. The included sample size therefore ranged from 3,250-3,257 (mean 3,254).

Whether a scratch was performed was imputed for 2 participants. The outcome live birth was imputed for 6 participants.

| **Supplementary Table S5.** Results of two-stage and one-stage IPD meta-analysis on all studies that shared IPD – Intention to treat analysis. | | | | | | | | | | | | | | | | | | | | | | | | | | | | | | | | | | | |
| --- | --- | --- | --- | --- | --- | --- | --- | --- | --- | --- | --- | --- | --- | --- | --- | --- | --- | --- | --- | --- | --- | --- | --- | --- | --- | --- | --- | --- | --- | --- | --- | --- | --- | --- | --- |
| **Outcome** | | **Studies** | | **Participants** | | **Method** | | **OR** | | **95% CI** | | | | | | | | | |  | **95% PI** | | | | | | | | | | **Intercept** | | **Trtm effect** | | **Significant adjustments** |
| Live birth | | 13 | | 4,112 | | Two-Stage | | 1.28 | | [ | | 1.02 | | | , | 1.61 | | ] | | * | [ | | 0.80 | | | , | 2.05 | | ] | | Fixed | | Random | | n.a. |
|  |  |  | |  | | One-Stage unadjusted | | 1.27 | | [ | | 1.04 | | | , | 1.54 | | ] | | * | [ | | 0.88 | | | , | 1.83 | | ] | | Random | | Random | | n.a. |
|  |  |  | |  | | One-Stage adjusted ^a^ | | 1.29 | | [ | | 1.02 | | | , | 1.64 | | ] | | * | [ | | 0.74 | | | , | 2.25 | | ] | | Random | | Random | | age, undergoing fertility treatment |
| Ongoing pregnancy | | 13 | | 4,112 | | Two-Stage | | 1.24 | | [ | | 0.99 | | | , | 1.54 | | ] | |  | [ | | 0.80 | | | , | 1.91 | | ] | | Fixed | | Random | | n.a. |
|  |  |  | |  | | One-Stage unadjusted | | 1.23 | | [ | | 1.01 | | | , | 1.50 | | ] | | * | [ | | 0.86 | | | , | 1.77 | | ] | | Random | | Random | | n.a. |
|  |  |  | |  | | One-Stage adjusted ^a^ | | 1.25 | | [ | | 0.98 | | | , | 1.59 | | ] | |  | [ | | 0.72 | | | , | 2.17 | | ] | | Random | | Random | | age, undergoing fertility treatment |
| Clinical pregnancy | | 13 | | 4,112 | | Two-Stage | | 1.28 | | [ | | 1.03 | | | , | 1.59 | | ] | | * | [ | | 0.85 | | | , | 1.92 | | ] | | Fixed | | Random | | n.a. |
|  |  |  | |  | | One-Stage unadjusted | | 1.26 | | [ | | 1.04 | | | , | 1.52 | | ] | | * | [ | | 0.97 | | | , | 1.63 | | ] | | Random | | Random | | n.a. |
|  |  |  | |  | | One-Stage adjusted ^a^ | | 1.30 | | [ | | 1.03 | | | , | 1.63 | | ] | | * | [ | | 0.80 | | | , | 2.11 | | ] | | Random | | Random | | age, undergoing fertility treatment, infertility duration |
| Miscarriage rate ^b^ | | 12 | | 1,278 – 1,285 | | Two-Stage | | 0.96 | | [ | | 0.67 | | | , | 1.39 | | ] | |  | [ | | 0.56 | | | , | 1.64 | | ] | | Fixed | | Random | | n.a. |
|  |  |  | |  | | One-Stage unadjusted | | 0.99 | | [ | | 0.73 | | | , | 1.35 | | ] | |  | [ | | 0.70 | | | , | 1.41 | | ] | | Random | | Random | | n.a. |
|  |  |  | |  | | One-Stage adjusted ^a^ | | 1.01 | | [ | | 0.72 | | | , | 1.42 | | ] | |  | [ | | 0.63 | | | , | 1.64 | | ] | | Random | | Random | | age |
| Miscarriage rate without Lee | | 11 | | 1,278– 1,281 | | Two-Stage | | 0.96 | | [ | | 0.65 | | | , | 1.42 | | ] | |  | [ | | 0.55 | | | , | 1.68 | | ] | | Fixed | | Random | | n.a. |
|  |  |  | |  | | One-Stage unadjusted | | 0.99 | | [ | | 0.73 | | | , | 1.34 | | ] | |  | [ | | 0.70 | | | , | 1.39 | | ] | | Random | | Random | | n.a. |
|  |  |  | |  | | One-Stage adjusted | | 1.01 | | [ | | 0.71 | | | , | 1.42 | | ] | |  | [ | | 0.61 | | | , | 1.65 | | ] | | Random | | Random | | none |
| * p < 0.05  OR: odds ratio; CI: confidence interval; PI: prediction interval; Trtm effect: treatment effect; n.a.: not applicable.  ^a^ Adjusted models are adjusted for age (grand mean-centered), body mass index (BMI) (grand mean-centered), duration of infertility (grand mean-centered), number of previous failed transfers, cause of infertility (unexplained = reference, male factor, other), and type of treatment (none = reference, fresh, frozen). Thus, the reference patient has average age, average BMI, average duration of infertility, and no previous failed transfers. Additionally, in adjusted models, the reference patient has a diagnose of unexplained infertility and has undergone no fertility treatment.  ^b^ The analyses for miscarriage rate are restricted to those participants who achieved a clinical pregnancy. Data on clinical pregnancy and miscarriage was imputed for 96 participants. Thus, the sample size for each imputation round varied between 1,278 and 1,285 participants (mean = 1,276). Studies that systematically did not record the outcome miscarriage were excluded (n=1). | | | | | | | | | | | | | | | | | | | | | | | | | | | | | | | | | | | |
| **Supplementary Table S6.** Results of two-stage and one-stage IPD meta-analysis on all studies that shared IPD – AT analysis. | | | | | | | | | | | | | | | | | | | | | | | | | | | | | | | | | | | |
| **Outcome** | **Studies** | | **Participants** | | **Method** | | **OR** | | **95% CI** | | | | | | | | | |  | **95% PI** | | | | | | | | | | **Intercept** | | **Trtm effect** | | **Significant adjustments** | |
| Live birth | 12 | | 3,954 | | Two-Stage | | 1.24 | | [ | | 0.99 | | , | 1.54 | | | ] | |  | [ | | 0.83 | | , | 1.84 | | | ] | | Fixed | | Random | | n.a. | |
|  |  | |  | | One-Stage unadjusted | | 1.22 | | [ | | 1.01 | | , | 1.47 | | | ] | | * | [ | | 0.90 | | , | 1.65 | | | ] | | Random | | Random | | n.a. | |
|  |  | |  | | One-Stage adjusted | | 1.22 | | [ | | 0.96 | | , | 1.54 | | | ] | |  | [ | | 0.73 | | , | 2.02 | | | ] | | Random | | Random | | age, undergoing fertility treatment, infertility duration | |
| Ongoing pregnancy | 12 | | 3,954 | | Two-Stage | | 1.20 | | [ | | 0.97 | | , | 1.48 | | | ] | |  | [ | | 0.82 | | , | 1.75 | | | ] | | Fixed | | Random | | n.a. | |
|  |  | |  | | One-Stage unadjusted | | 1.19 | | [ | | 0.99 | | , | 1.43 | | | ] | |  | [ | | 0.89 | | , | 1.58 | | | ] | | Random | | Random | | n.a. | |
|  |  | |  | | One-Stage adjusted | | 1.18 | | [ | | 0.94 | | , | 1.49 | | | ] | |  | [ | | 0.73 | | , | 1.92 | | | ] | | Random | | Random | | age, undergoing fertility treatment, infertility duration | |
| Clinical pregnancy | 12 | | 3,954 | | Two-Stage | | 1.21 | | [ | | 1.01 | | , | 1.46 | | | ] | | * | [ | | 0.94 | | , | 1.56 | | | ] | | Fixed | | Random | | n.a. | |
|  |  | |  | | One-Stage unadjusted | | 1.20 | | [ | | 1.04 | | , | 1.40 | | | ] | | * | [ | | 1.02 | | , | 1.42 | | | ] | | Random | | Random | | n.a. | |
|  |  | |  | | One-Stage adjusted | | 1.20 | | [ | | 0.96 | | , | 1.51 | | | ] | |  | [ | | 0.80 | | , | 1.81 | | | ] | | Random | | Random | | age, undergoing fertility treatment, infertility duration | |
| Miscarriage rate | 11 | | 1,216-1,231 | | Two-Stage | | 0.97 | | [ | | 0.67 | | , | 1.40 | | | ] | |  | [ | | 0.57 | | , | 1.64 | | | ] | | Fixed | | Random | | n.a. | |
|  |  | |  | | One-Stage unadjusted | | 0.99 | | [ | | 0.73 | | , | 1.35 | | | ] | |  | [ | | 0.72 | | , | 1.35 | | | ] | | Random | | Random | | n.a. | |
|  |  | |  | | One-Stage adjusted | | 0.99 | | [ | | 0.71 | | , | 1.39 | | | ] | |  | [ | | 0.65 | | , | 1.52 | | | ] | | Random | | Random | | age | |
| Miscarriage rate without Lee | 10 | | 1,212-1,227 | | Two-Stage | | 0.97 | | [ | | 0.65 | | , | 1.43 | | | ] | |  | [ | | 0.56 | | , | 1.68 | | | ] | | Fixed | | Random | | n.a. | |
|  |  | |  | | One-Stage unadjusted | | 0.99 | | [ | | 0.72 | | , | 1.34 | | | ] | |  | [ | | 0.72 | | , | 1.34 | | | ] | | Random | | Random | | n.a. | |
|  |  | |  | | One-Stage adjusted | | 0.98 | | [ | | 0.70 | | , | 1.38 | | | ] | |  | [ | | 0.64 | | , | 1.51 | | | ] | | Random | | Random | | age | |
| * p < 0.05  AT: as treated; OR: odds ratio; CI: confidence interval; PI: prediction interval; Trtm effect: treatment effect; n.a.: not applicable. 12 studies (3,954 participants).  Adjusted models are adjusted for age (grand mean-centered), body mass index (BMI) (grand mean-centered), duration of infertility (grand mean-centered), number of previous failed transfers, cause of infertility (unexplained = reference, male factor, other), and type of treatment (none = reference, fresh, frozen). Thus, the reference patient has average age, average BMI, average duration of infertility, and no previous failed transfers. Additionally, in adjusted models, the reference patient has a diagnose of unexplained infertility and has undergone no fertility treatment.  The analyses for miscarriage rate are restricted to the studies that recorded data on miscarriage (11 studies, 3,855 participants), and to those participants who achieved a clinical pregnancy (1,216 to 1,231, average 1,218, out of the 3,855 participants). Data on clinical pregnancy was imputed for 96 participants; data on miscarriage was imputed for 2 participants. | | | | | | | | | | | | | | | | | | | | | | | | | | | | | | | | | | | |

| **Supplementary Table S7** Results of two-stage and one-stage IPD meta-analysis on all studies that shared IPD – AT+ET analysis. | | | | | | | | | | | | | | | | | | | |
| --- | --- | --- | --- | --- | --- | --- | --- | --- | --- | --- | --- | --- | --- | --- | --- | --- | --- | --- | --- |
| **Outcome** | **Studies** | **Participants** | **Method** | **OR** | **95% CI**. | | | | |  | | **95% PI** | | | | | **Intercept** | **Trtm. effect** | **Significant adjustments** |
| Live birth | 12 | 3,250-3,257 | Two-Stage | 1.25 | [ | 0.97 | , | 1.60 | ] |  | | [ | 0.73 | , | 2.12 | ] | Fixed | Random | n.a. |
|  |  |  | One-Stage unadjusted | 1.24 | [ | 0.99 | , | 1.54 | ] |  | | [ | 0.81 | , | 1.89 | ] | Random | Random | n.a. |
|  |  |  | One-Stage adjusted | 1.25 | [ | 0.99 | , | 1.57 | ] |  | | [ | 0.77 | , | 2.03 | ] | Random | Random | age, infertility duration |
| Ongoing pregnancy | 12 | 3,250-3,257 | Two-Stage | 1.21 | [ | 0.95 | , | 1.55 | ] |  | | [ | 0.72 | , | 2.04 | ] | Fixed | Random | n.a. |
|  |  |  | One-Stage unadjusted | 1.20 | [ | 0.97 | , | 1.49 | ] |  | | [ | 0.79 | , | 1.82 | ] | Random | Random | n.a. |
|  |  |  | One-Stage adjusted | 1.21 | [ | 0.96 | , | 1.52 | ] |  | | [ | 0.76 | , | 1.92 | ] | Random | Random | age, infertility duration |
| Clinical pregnancy | 12 | 3,250-3,257 | Two-Stage | 1.24 | [ | 0.99 | , | 1.56 | ] |  | | [ | 0.81 | , | 1.91 | ] | Fixed | Random | n.a. |
|  |  |  | One-Stage unadjusted | 1.23 | [ | 1.00 | , | 1.51 | ] | * | | [ | 0.88 | , | 1.72 | ] | Random | Random | n.a. |
|  |  |  | One-Stage adjusted | 1.23 | [ | 1.00 | , | 1.52 | ] | * | | [ | 0.86 | , | 1.77 | ] | Random | Random | age, infertility duration, treatment type |
| Miscarriage rate ^a^ | 11 | 1,186-1,187 | Two-Stage | 0.97 | [ | 0.68 | , | 1.38 | ] |  | [ | | 0.66 | , | 1.43 | ] | Fixed | Random | n.a. |
|  |  |  | One-Stage unadjusted | 0.98 | [ | 0.72 | , | 1.34 | ] |  | | [ | 0.72 | , | 1.34 | ] | Random | Random | n.a. |
|  |  |  | One-Stage adjusted | 0.99 | [ | 0.72 | , | 1.38 | ] |  | | [ | 0.69 | , | 1.42 | ] | Random | Random | age |
| Miscarriage rate without Lee | 10 | 1,182-1,183 | Two-Stage | 0.97 | [ | 0.67 | , | 1.42 | ] |  | | [ | 0.64 | , | 1.47 | ] | Fixed | Random | n.a. |
|  |  |  | One-Stage unadjusted | 0.97 | [ | 0.71 | , | 1.33 | ] |  | | [ | 0.71 | , | 1.33 | ] | Random | Random | n.a. |
|  |  |  | One-Stage adjusted | 0.98 | [ | 0.70 | , | 1.37 | ] |  | | [ | 0.68 | , | 1.42 | ] | Random | Random | age |
| * p < 0.05  AT+ET: as treated with embryo transfer; OR: odds ratio; CI: confidence interval; PI: prediction interval; trtm. Effect: treatment effect; n.a.: not applicable.  Number of included studies: 12. Data was restricted to those receiving an embryo transfer. Data on receiving an embryo transfer was imputed for 19 participants, therefore the number of included participants for the analyses varied between 3,250 and 3,257 (mean=3,254).  Adjusted models are adjusted for age (grand mean-centered), body mass index (BMI) (grand mean-centered), duration of infertility (grand mean-centered), number of previous failed transfers, cause of infertility (unexplained = reference, male factor, other), and type of treatment (none = reference, fresh, frozen). Thus, the reference patient has average age, average BMI, average duration of infertility, no previous failed transfers, a diagnosis of unexplained infertility, and has undergone no fertility treatment/embryo transfer.  ^a^ Analysis of miscarriage was restricted to participants who reached a clinical pregnancy. Data on clinical pregnancy was imputed for 96 participants, and on miscarriage for 2 participants. The number of included participants ranged from 1,186-1,187 (mean 1,186). | | | | | | | | | | | | | | | | | | | |

**Supplementary Figure S3:** Forest plot of two-stage Intention to Treat analysis, outcome: Ongoing Pregnancy.

OR, odds ratio. 95% CI, 95% confidence interval. 13 studies (4,112 participants). Shown event numbers are observed values only. The per-study and pooled odds ratios are based on imputed data. Outcomes were imputed for 57 participants in the scratch group and 68 in control (fraction of missing outcome data 3.0%).

**Supplementary Figure S4:** Forest plot of two-stage As Treated analysis, outcome: Ongoing Pregnancy.

AT: as treated; OR: odds ratio; 95% CI: 95% confidence interval. 12 studies (3,954 participants).

Shown event numbers are observed values only. The per-study and pooled odds ratios are based on imputed data. Whether a scratch was performed was imputed for 2 participants.

The outcome ongoing pregnancy was imputed for 114 participants (fraction of missing outcome data 2.9%).

**Supplementary Figure S5:** Forest plot of two-stage As Treated with Embryo Transfer analysis, outcome: Ongoing Pregnancy.

AT+ET; as treated with embryo transfer; OR, odds ratio; 95% CI: 95% confidence interval. 12 studies.

Shown event numbers are observed values only. The per-study and pooled odds ratios are based on imputed data.

Analysis was restricted to those patients receiving an embryo transfer. Data on embryo transfer was imputed for 19 participants. The included sample size therefore ranged from 3,250-3,257 (mean 3,254).

Whether a scratch was performed was imputed for 2 participants. The outcome ongoing pregnancy was imputed for 21 participants.

**Supplementary Figure S6:** Forest plot of two-stage Intention to Treat analysis, outcome: Clinical Pregnancy.

OR, odds ratio. 95% CI, 95% confidence interval. 13 studies (4,112 participants). Shown event numbers are observed values only. The per-study and pooled odds ratios are based on imputed data. Outcomes were imputed for 42 participants in the scratch group and 54 in control (fraction of missing outcome data 2.3%).

**Supplementary Figure S7:** Forest plot of two-stage As Treated analysis, outcome: Clinical Pregnancy.

AT: as treated; OR: odds ratio; 95% CI: 95% confidence interval. 12 studies (3,954 participants). Shown event numbers are observed values only. The per-study and pooled odds ratios are based on imputed data. Whether a scratch was performed was imputed for 2 participants.

The outcome clinical pregnancy was imputed for 96 participants (fraction of missing outcome data 2.4%).

**Supplementary Figure S8:** Forest plot of two-stage As Treated with Embryo Transfer analysis, outcome: Clinical Pregnancy.

AT+ET; as treated with embryo transfer; OR, odds ratio; 95% CI: 95% confidence interval. 12 studies.

Shown event numbers are observed values only. The per-study and pooled odds ratios are based on imputed data.

Analysis was restricted to those patients receiving an embryo transfer. Data on embryo transfer was imputed for 19 participants. The included sample size therefore ranged from 3,250-3,257 (mean 3,254).

Whether a scratch was performed was imputed for 2 participants. The outcome clinical pregnancy was imputed for 3 participants.

**Supplementary Figure S9:** Forest plot of two-stage Intention to Treat analysis, outcome: Miscarriage.

OR, odds ratio. 95% CI, 95% confidence interval. 11 studies.

Shown event numbers are observed values only. The per-study and pooled odds ratios are based on imputed data.

Analysis was restricted to participants achieving a clinical pregnancy. Data on clinical pregnancy was imputed for 96 participants. Therefore, the sample size ranged from 1,278-1,285 (mean 1,280) participants.

The outcome miscarriage was also imputed for 96 participants.

**Supplementary Figure S10**: Forest plot of two-stage As Treated analysis, outcome: Miscarriage.

AT: as treated; OR: odds ratio; 95% CI: 95% confidence interval. 11 studies.

Shown event numbers are observed values only. The per-study and pooled odds ratios are based on imputed data. Whether a scratch was performed was imputed for 2 participants.

Analysis was restricted to participants achieving a clinical pregnancy. Data on clinical pregnancy was imputed for 96 participants. Therefore, the sample size ranged from 1,278-1,285 (mean 1,280) participants.

The outcome miscarriage was imputed for 98 participants.

**Supplementary Figure S11:** Forest plot of two-stage As Treated with Embryo Transfer analysis, outcome: Miscarriage.

AT+ET; as treated with embryo transfer; OR, odds ratio; 95% CI: 95% confidence interval. 11 studies.

Shown event numbers are observed values only. The per-study and pooled odds ratios are based on imputed data. Whether a scratch was performed was imputed for 2 participants.

Analysis was restricted to participants achieving a clinical pregnancy. Data on clinical pregnancy was imputed for 96 participants. Therefore, the sample size ranged from 1,186-1,187 (mean 1,186) participants.

The outcome miscarriage was imputed for 98 participants.

| **Supplementary Table S8.** Numbers of events for the adverse outcomes ‘ectopic pregnancy’ and ‘ongoing multiple pregnancy’. | | | | |
| --- | --- | --- | --- | --- |
|  | | **Number of events** | **Number of missing values** | ***n*** |
| **Intention to treat** | |  |  |  |
| **Outcome: Ectopic pregnancy** | |  |  |  |
|  | Control group | 7 | 260 | 2,053 |
|  | Scratch group | 8 | 208 | 2,059 |
| **Outcome: Ongoing multiple pregnancy** | |  |  |  |
|  | Control group | 45 | 295 | 2,053 |
|  | Scratch group | 53 | 278 | 2,059 |
| **As treated** | |  |  |  |
| **Outcome: Ectopic pregnancy** ^a^ | |  |  |  |
|  | Control group | 8 | 206 | 2,043 |
|  | Scratch group | 7 | 154 | 1,909 |
| **Outcome: Ongoing multiple pregnancy** ^a^ | |  |  |  |
|  | Control group | 42 | 301 | 2,043 |
|  | Scratch group | 41 | 271 | 1,909 |
| Observed data only. For Intention to treat: all studies were included (13 studies; 4,112 participants). For As treated: all studies except Nastri et al. were included (12 studies; 3,954 participants).  ^a^ Data on whether a scratch was performed was imputed for 2 participants: 1 in the scratch and 1 in the control group. The participant in the scratch group did not have an ectopic pregnancy or ongoing multiple pregnancy. For the participant in the control group, data on ectopic pregnancy and ongoing multiple pregnancy was missing. Due to the low number of events, no formal comparative analysis was performed. | | | | |

**Supplementary Figure S12:** Forest plot of two-stage Intention to Treat analysis, outcome Live Birth.

*Comparison of studies with different timing and different menstrual cycle type.*

LB, live birth. Total, total number of randomized participants. OR, odds ratio. 95%CI, 95% confidence interval. OCP, oral contraceptive pill.

13 studies, 4,112 participants.

Events of LB and totals are based on observed data. The calculated ORs are based on imputed data.

Luteal phase, natural cycle: studies that performed one or two scratches in the luteal phase of a natural pre-transfer cycle.

Luteal phase, OCP cycle: studies that performed one scratch at 4-14 days prior to the start of ovarian stimulation in an oral contraceptive pill cycle.

Other: other timing of the scratch. This includes unspecified timing in the pre-transfer cycle and scratching in the follicular phase of the transfer cycle, and includes both natural and oral contraceptive pill cycles.
